# New Light on the Evolutionary History of the Common Goby (*Pomatoschistus microps*) with an Emphasis on Colonization Processes in the Mediterranean Sea

**DOI:** 10.1371/journal.pone.0091576

**Published:** 2014-03-19

**Authors:** Christelle Tougard, Joy Folly, Patrick Berrebi

**Affiliations:** Institut des Sciences de l’Evolution de Montpellier, UMR CNRS 5554, Montpellier, France; University of Gothenburg, Sweden

## Abstract

Through the study of the phylogeographic structure and demographic history of the common goby, *Pomatoschistus microps*, the influence of Quaternary climatic changes on the evolutionary history of coastal and marine fishes is investigated. Because of its sedentary life cycle in Mediterranean lagoons, it is also a good model to study more specifically if the formation of lagoons during the Holocene had an impact on population structure and demography. Mitochondrial sequences of Northeastern Atlantic and Western Mediterranean specimens were used for phylogenetic reconstructions as well as divergence time estimates, demographic history and population structure analyses. *Pomatoschistus microps* was a highly supported monophyletic clade including four lineages. It may have appeared 77,000 yr ago, and the divergence of its lineages likely occured shortly thereafter (between 61,000 and 54,000 yr). Most lineages had polytomic topologies, low nucleotide diversity and demographic analyses providing evidence of population expansion. Each lineage was characterized by a large number of private haplotypes. Most haplotypes found in Mediterranean localities were endemic, and one was dominant. Complex reticulated relationships connecting North European, Atlantic and Mediterranean haplotypes were observed. Moderate to high population structure was underlined. Contrary to previous published studies, no significant differentiation was observed between Atlantic and Mediterranean populations, indicating that the Gibraltar Strait is not a phylogeographic break for *P. microps*. Indeed, molecular dating combined with the tree topologies, phylogeographic and demographic analyses as well as high haplotype diversity underline a recent and rapid population divergence during the last glacial. However, population structure indicates that differentiation is an ongoing process. From an ancestral population trapped in the Atlantic, this goby colonized first northern Europe and later the Mediterranean Sea. Shared haplotypes could have dispersed in the western Mediterranean basin before the lagoon formation, while most private haplotypes, evidencing a recent isolation, probably diverged in lagoons after their closure.

## Introduction

The Mediterranean Sea (MS) is a region of high biodiversity and endemism [1], [2], [3], [4]. This biota clusters species from different biogeographical origins: (1) temperate Atlantic-Mediterranean species; (2) cosmopolitan/panoceanic species; (3) endemic species, notably those with a Miocene or Pliocene origin; (4) subtropical Atlantic species (Quaternary interglacial remnants); (5) boreal Atlantic species (Quaternary glacial remnants); (6) Red Sea migrants; (7) eastern Atlantic migrants [1]. In a general way, the central parts of the MS are characterized by “typical” Mediterranean species. The Alboran Sea, located immediately east of the Gibraltar Strait, hosts species with Atlantic affinities, whereas the Levant Sea concentrates species of the Red Sea after the opening of the Suez Canal in 1869 [1]. The combined effects of MS warming and anthropogenic introduction of invasive and exotic species are reshaping the MS biodiversity, leading gradually to the “tropicalization” of the Mediterranean coastal and marine fauna and flora [3], [4]. Despite these recent changes, the origins of the Mediterranean coastal and marine biodiversity are rooted in the complex and tormented geological history of the Mediterranean Sea.

This history is the result of diverse tectonic events spanning some 250 Myr, from the late Triassic to the Quaternary [5]. The ancient history is related to a large and complex oceanic domain, the Tethys (Palaeo- and Neotethys), whereas the recent history is associated with the opening of the Atlantic Ocean and dominated by the Afro-Arabian and Eurasian continental plate collision [6]. During the Messinian Salinity Crisis (MSC; 6.14–5.96 Myr) [7], the desiccation of the MS by the reduction of water exchanges with the Atlantic caused a major extinction of deep-water fauna [1], [7], [8], [9]. After the refilling of the MS by Atlantic waters, the renewal of the marine Mediterranean biota was characterized by a strong increase of modern component [10]. Recurrent, short periods of separation between the Atlantic and Mediterranean waters also occurred during cyclic Quaternary ice ages [11]. Temperature, salinity, water circulation and sea levels were notably impacted by these Quaternary climatic changes [11], [12], [13], [14]. Different immigration waves of Atlantic boreal or subtropical species occurred in alternation during glacial and interglacial periods, respectively [1]. The Holocene deglacial rapid sea-level rise combined with the decrease of erosion and of sediment transport by rivers favoured the establishment of lagoons on Mediterranean coastal areas: for instance, lagoons of the Golfe du Lion (GDL), France [15], [16]; Orbetello and Lesina lagoons, Italy [17], [18]; Albufereta lagoon, Spain [19]; Ververonda lagoon, Greece [20].

The Gobiidae is one of the most speciose families of fish with nearly 2,000 species living in tropical or temperate seas worldwide [21], [22], [23]. Gobies inhabit shallow marine, brackish water, estuarine or freshwater environments [21], [23]. Along the Eastern Atlantic and Mediterranean coasts, *Pomatoschistus* is the most diverse genus with approximately 12 species (http://fishbase.mnhn.fr). The origin of Gobiinae, to which the genus *Pomatoschistus* belongs to, likely lies within the MS, where the earliest fossil remains of gobies were discovered in Eocene sediments [24]. The Mediterranean desiccation and then re-opening during the MSC had a major impact on *Pomatoschistus* speciation quite like the climatic changes of the Pleistocene [25]. Most *Pomatoschistus* species are Mediterranean endemics, few of them inhabiting also the Eastern Atlantic. Because *Pomatoschistus* is a paraphyletic genus, phylogenetic relationships between *Pomatoschistus* species remain unclear [25], [26]. Studies focused on species distributed in the MS (*P. marmoratus* and *P. tortonesei*) underlined a genetic differentiation between the western and eastern Mediterranean basins [27], [28]. The Siculo-Tunisian Strait provides a barrier to dispersal, and thus to gene flow. Some species (*P. minutus* and *P. microps*), with an Atlantic-Mediterranean distribution, show a high degree of genetic differentiation between Atlantic and Mediterranean populations. The Almería-Oran Oceanic Front, in the Alboran Sea, seems to be a barrier to dispersal [29], [30], [31], [32]. In most studies, a restricted number of populations or of geographic areas were investigated to reconstruct the evolutionary history of Mediterranean populations. The potential structuring role played by the formation of lagoons on these populations was never discussed.

The common goby, *Pomatoschistus microps*, is a small euryhaline fish found in estuaries along the Atlantic coasts from Norway to Morocco, including Baltic Sea [21], [33], [34], [35]. In the western Mediterranean basin, this fish widely occurs in coastal lagoons [21], [36]. It is a sedentary and annual species in the MS, while it is a migratory species able to live up to two years in Northeastern Atlantic [33], [36]. Despite a pelagic larval phase, the common goby is only able to migrate over short distances [36]. Mediterranean and Atlantic populations display also differences in their growth rate, life cycle, size, sex ratio and spawning time [36]. It is found in sympatry with *P. marmoratus* in some lagoons of the GDL (*e.g.* Vaccarès lagoon complex), where they hybridise, producing fertile offspring [37]. *P. microps* is phylogenetically closely related to the endemic Mediterranean species *P. tortonesei*, *P. knerii* and *P. marmoratus* [25], [27]. It is thus supposed to be of Mediterranean origin [30].

In the present work, we use the common goby as a model to infer colonization processes of coastal marine fishes in the western Mediterranean Sea. Because of its sedentary life cycle in the lagoons of the MS, it is a good model to study more specifically if the formation of lagoons during the Holocene had an impact on population structure and demography. Mitochondrial sequences from the control region (CR) and cytochrome *b* gene (cytb) were used to investigate its phylogeographic structure and demographic history. Eighteen Mediterranean (6 from Spain and 12 from France) and 15 Atlantic (1 in United Kingdom, 1 in Ireland, 5 in France and 8 in Portugal) localities were considered here. Biogeographic traits (present-day geographic distribution, habitat preference and life-history traits) were also integrated in this study.

## Materials and Methods

### Sampling

The studied gobies were provided by professional fishermen as they are commonly trapped in fishing nets with fishes of commercial interest. For this reason, an ethical approval is not required.

**Figure 1:**
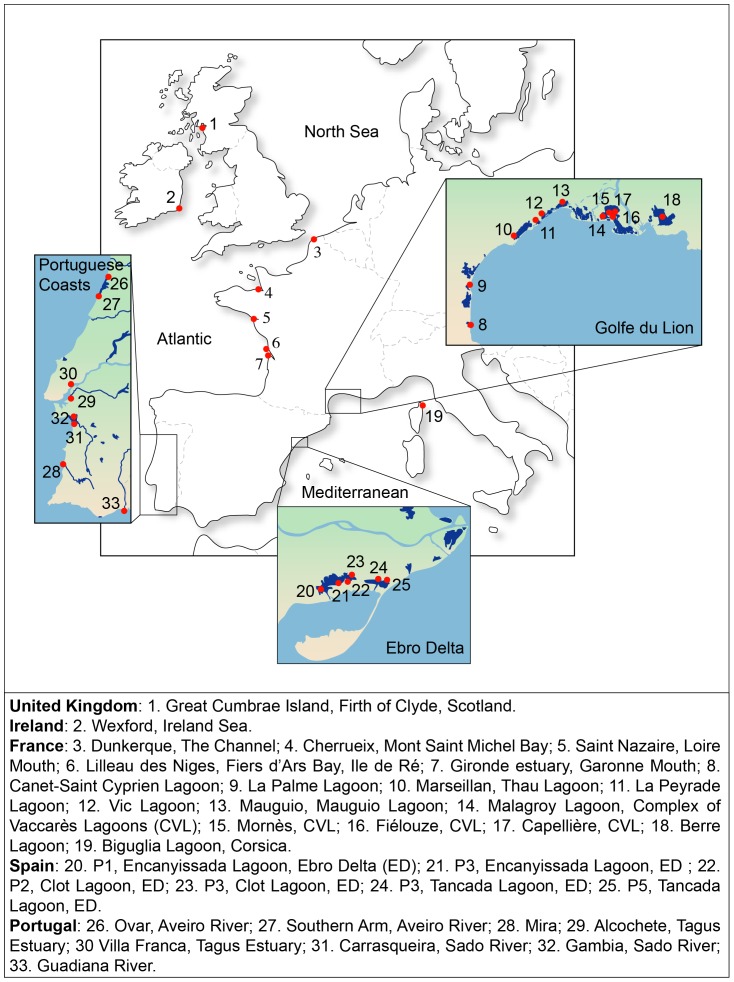
Locality distribution for the common goby, *Pomatoschistus microps*, in the Northeastern Atlantic Ocean and Mediterranean Sea. Details about localities and samples are in Table S1.

The dataset includes, respectively, 239 and 217 original partial sequences of the control region and cytochrome *b* gene from the whole distribution of the common goby, *Pomatoschistus microps*. This dataset was completed by original sequences of *P. marmoratus*, *P. tortonesei*, *P. minutus*, *P. canestrinii* and *Knipowitschia panizzae*, as well as GenBank sequences of *P. lozanoi* and *K. caucasica*. All these latter species of *Pomatoschistus* and *Knipowitschia* were used as outgroup. Details about the samples are given in Fig. 1 and Table S1.

### DNA Sequencing and Alignment

DNA extractions were performed from 96% ethanol-preserved fin or muscle tissue using the CHELEX method [38]. Fragments of mitochondrial DNA (mtDNA) were PCR-amplified with conserved or specific primers: for CR, DL1 5′-ACCCCTGGCTCCCAAAGC-3′ [39] and Pom2R 5′-CTGCACTGTGAGATGCCA-3′: for cytb, PomCB1F 5′-TTCCCCTTTGTAGTACTAGCAG-3′ and GobCB2R 5′-GGGAGGRDTTTAACCTCCG-3′. Amplification conditions consisted of an initial DNA denaturation at 94°C for 5 min, followed by 35 cycles including a denaturation at 94°C for 45 s, an annealing at 50°C for 1 min, an extension at 72°C for 2 min, and a final extension at 72°C for 10 min. Sequences produced by a private company (Macrogen Inc., Seoul, South Korea) were obtained on both strands to confirm polymorphic sites. They were aligned by hand using MEGA v5.05 [40].

### Phylogenetic Inferences and Divergence Time Estimates

Phylogenetic analyses were conducted on both separate and concatenated genes. Topological congruence between CR and cytb was evaluated using PAUP*4.010b « Partition-Homogeneity Test » option with 1000 replicates [41]. Two probabilistic methods were carried out for phylogenetic tree reconstructions: a maximum-likehood (ML) method with PhyML v2.4.5 [42] and a Bayesian inference (BI) with MrBayes v3.1.2 [43]. Appropriate models of sequence evolution were determined using Modeltest v3.7 [44] and MrModeltest v2.3 [45], respectively. ML reconstructions were conducted under the GTR model [46] with a proportion of invariable sites (I) and a gamma distribution (G). Node robustness was estimated by ML bootstrap percentages (BP) after 1000 pseudo-replicates. In BI, the best-fitting models of sequence evolution were, respectively for the first, second and third codon positions, the GTR+I+G, HKY+I+G [47] and F81+I+G [48] models. Node robustness was estimated with Bayesian posterior probabilities (PP) obtained from the 50% majority rule consensus of trees, and after discarding the first 25,000 trees as burn-in. Three independent runs of five incrementally heated Monte Carlo Markov chains were performed with trees sampled every 100^th^ generation for 5,000,000 generations. ML and BI topologies as well as lineage alternative topologies were compared with the test of Shimodaira & Hasegawa [49] as implemented in PAUP.

Substitution rates among sequences were compared using the relative rate test as implemented in RRTree [50]. Distances were computed for the concatenated sequences with Kimura’s two-parameter distance. Molecular dating of *P. microps* lineage divergence was calculated with the BaseML program implemented in the PAML package v4.5 [51] using a local molecular clock (*i.e*. a clock assuming the same substitution rate for closely related group and different rates in other parts of the tree) [52] and the ML tree obtained with PhyML. Different rates were assigned to slower and faster evolving species. The date of 6.05±0.09 Myr (between 6.14 and 5.96 Myr) [7] for the isolation of the Mediterranean Sea (and the subsequent origin of the sand goby freshwater lifestyle) [25] was used as calibration point.

### Genetic Variability and Population Structure

Genetic distance was estimated within and between *P. microps* lineages by the uncorrected *p*-distance with MEGA. The number of haplotypes (*nh*) per lineage, the nucleotide (*π*) and haplotype (*h*) diversities as well as the average number of nucleotide differences between two sequences (*k*) were obtained for separate and concatenated genes using DnaSP v 5.10.01b [53].

Lineage demographic history was investigated with DnaSP from three neutrality tests (Tajima’s *D* statistic, Fu’s *F*s and *R*_2_ test) [54], [55], [56]. Significant negative values can be expected in cases of population expansion (*p*<0.05). Mismatch distribution analyses (*i.e.* the frequency distribution of pairwise differences between individuals) [57] were also carried out. In this case, demographic stability is illustrated by multimodal distributions, while a unimodal pattern is consistent with sudden expansion [58]. Observed and expected distributions were compared with the sum of squared deviations (*SSD*) and with the raggedness index (*r*) of Harpending [59] as implemented by Arlequin v3.5.1.2 [60]. Hypothesis of demographic expansion is then rejected for *p*-values <0.05.

Population structure was evaluated by a hierarchical analysis of molecular variance (AMOVA) [61] as implemented by Arlequin. Φ-statistics were used to test significant partitions of genetic variance among several groups: 1) all *P. microps* lineages in one panmictic group; 2) Mediterranean vs Atlantic; 3) Mediterranean vs Atlantic vs northern Europe; 4) Spanish vs French Mediterranean populations. A non-metric multidimensional scaling analysis (NMDS) was performed using xlstat 4.05 (Addinsoft, hhttp://www.xlstat.com). The NMDS is based on the pairwise *F*_ST_ genetic distances calculated in Arlequin. A median-joining network of all *P. microps* haplotypes was constructed with Network v4.5.1.6 (http://www.fluxus-engineering.com/sharenet.htm) [62].

## Results

Except for phylogenetic inferences, all results presented here are based on the concatenated dataset (CR+cytb). The Atlantic1 (A1) lineage includes mainly Atlantic Portuguese and French individuals, while the Atlantic2 (A2) characterizes exclusively Atlantic Portuguese and French individuals. The North European (NE) and Mediterranean (M) lineages cluster mostly individuals from northern Europe (northern France, Ireland and Scotland) and the Mediterranean Sea, respectively.

### Phylogenetic Analyses and Molecular Dating

All new sequences were deposited in the EMBL database under accession numbers HF969335–HF969861.

The alignment of the control region represents 473 positions and 159 phylogenetically informative sites (PIS). ML and BI analyses provided different tree topologies (Fig. S1 and S2). Comparing these topologies with the SH test [49], the BI tree appears significantly worse than the ML tree at the 5% confidence level (*P* = 0.000). Though the monophyly of *P. microps* is supported by 97% BP in ML and 0.83 PP in BI, discrepancies are in intraspecific tree topologies only. In the ML tree, some individuals from the Atlantic French and Portuguese coasts constitute the first offshoot, while the other individuals are clustered in three *microps* lineages, M, NE and Atlantic (mostly Atlantic Portuguese individuals), but with low supports (BP<50%). The BI tree is a polytomy and it includes *P. tortonesei* individuals among *P. microps*.

The alignment of the cytochrome *b* gene is 565 nucleotides long and it has 188 PIS. Despite some differences in the branching patterns (Fig. S3), the SH test did not reveal significant incoherence between the ML and BI approaches (*P* = 0.064). The monophyly of *P. microps* is also well supported (BP = 100%; PP = 0.91). M, NE and A2 lineages are well identified with both approaches and they have high supports in BI (respectively, PP = 0.84, 0.98 and 0.91). The A2 lineage has also moderate ML bootstrap percentages (BP = 59%).

**Figure 2:**
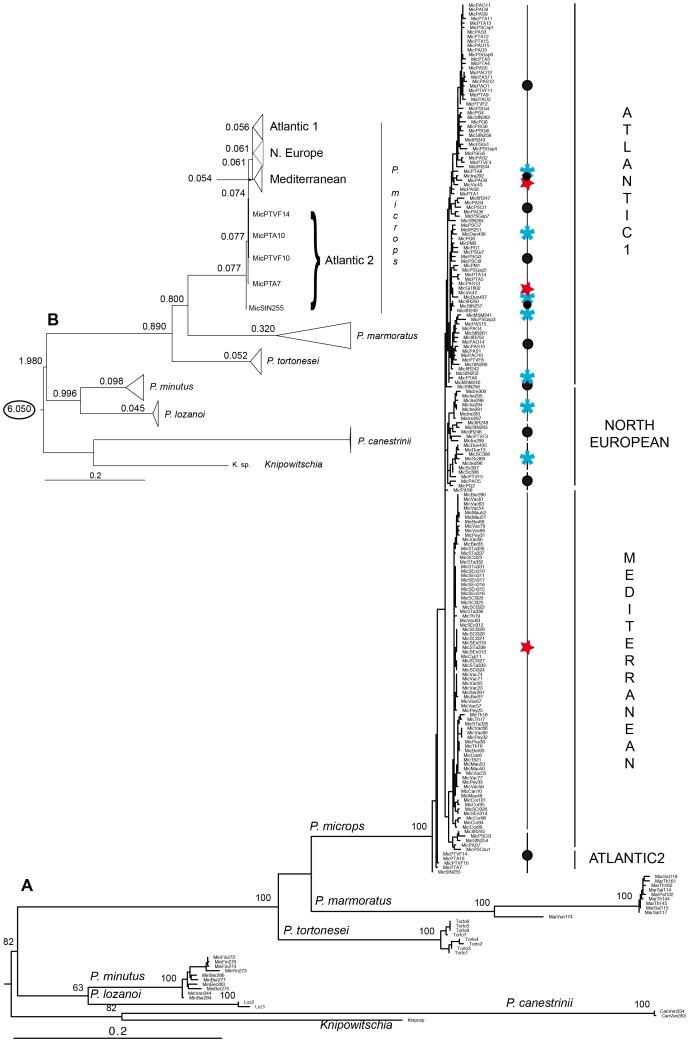
Maximum-likelihood tree reconstructed from concatenated control region and cytochrome *b* gene sequences from *Pomatoschistus microps*. **A**. Individual labels are detailed in Table S1. Individuals with • are from an Atlantic locality, whereas individuals with blue * and red star are, respectively, from northern Europe and Mediterranean localities. Numbers at nodes are for ML bootstrap percentages (≥50%). **B**. Numbers at nodes are for divergence times (Myr) estimated with PAML and based on a geologic calibration point, 6.05±0.09 Myr (between 6.14 and 5.96 Myr) [7]. For both figures, *P. microps* lineages are indicated on the right.

Discrepancies exist between the CR and cytb tree topologies, probably due to their different evolutionary rates. However, the “Partition Homogeneity Test” performed with PAUP did not reveal significant incongruence between CR and cytb (0.80 =  *P*>0.05). For this reason, the CR and cytb datasets were concatenated. The CR+cytb dataset includes 194 sequences of *P. microps*. Phylogenetic analyses were performed on 1038 sites (346 PIS). At the 5% confidence level, the BI tree appears significantly worse than the ML tree (*P* = 0.007) using the SH test (Fig. 2A and Fig. S4). *P. microps* is monophyletic with high support (BP = 100%; PP = 0.87). In the concatenated ML tree, three lineages, M, A1 and NE, are identified. Individuals from the A2 lineage constitute the first offshoot of the *P. microps* clade. In the concatenated BI tree, the M and NE lineages are well supported (PP = 0.84 and 0.91, respectively). Investigating alternative topologies with the SH test, over the 105 topologies among the five tested clades (M, A1, A2, NE and Outgroup), none was significantly worse than the highest likelihood tree (all *P*’s >0.189; Table S2). The best ML tree (Tree 1; Table S2) considers the NE lineage as outgroup, but also the A2 individuals and the outgroup species as sister groups. As such, it differs from the trees shown in Fig. 2A (ML) and Fig. S4 (BI) not significantly worse (Tree 6 with *P* = 0.966; Table S2).

Because of the result of the SH test performed on the ML and BI trees, no method involving a Bayesian approach (neither Bayesian coalescent analysis for molecular dating estimates nor Bayesian Skyline Plot for demographic history) was used in the following analyses. For this reason, the relative rate test was used to determine whether the observed substitution rates (r) were constant between the *Pomatoschistus* species. Each species was tested relative to the other species using *Knipowitschia* as outgroup. *P. minutus* and *P. lozanoi* appeared to evolve with a significantly slower rate (7 10^−6^<*P*<0.0124) than the other *Pomatoschistus* species. Concurrently, *P. tortonesei* was characterized by a faster evolutionary rate (0.0003<*P*<0.0144). For these reasons, a local molecular clock model [52] as implemented in PAML and six local rates (0.001< r <3.123) were used to estimate divergence times. Based on a calibration point of 6.05±0.09 Myr, *P. microps* may have appeared approximately 77,000 years ago (Weichselian period). The divergence of the different lineages seemed to occur shortly after (between 61,000 and 54,000 yr). Divergence time estimates for *microps* lineages are indicated at nodes in Fig. 2B.

### Genetic Diversity and Structure

Genetic distance within and between each *P. microps* lineages (Table 1) was estimated, respectively: from 0.003±0.001 (A2) to 0.011±0.002 (A1); from 0.013±0.003 (A2/M) to 0.016±0.003 (A1/M and NE; NE/M). The nucleotide diversity was heterogeneous (A2 = 0.25%<π<A1 = 1.03%), whereas the haplotype diversity was relatively high and homogeneous for all lineages (A2 = 0.900< *h*<A1 = 0.995) (Table 2). The average number of pairwise nucleotide differences is high for most lineages (5.326< *k* <10.625) except for the A2 lineage (*k* = 2.600). All these analyses indicate that some Atlantic lineages (NE and especially A1) have the highest genetic diversity, while the A2 had the lowest.

**Table 1:** Genetic distance within and between the *Pomatoschistus microps* lineages and the species used as outgroup.

	Distance (SE)^a^	M	NE	A2	A1	Pt	Pma	Pmi	Pl	Pc	K
Mediterranean	0.005 (0.001)	–	0.003^c^	0.003	0.003	0.010	0.011	0.011	0.012	0.012	0.012
N. European	0.010 (0.002)	0.016^b^	–	0.003	0.003	0.011	0.012	0.011	0.012	0.012	0.012
Atlantic2	0.003 (0.001)	0.013	0.015	–	0.003	0.011	0.012	0.011	0.012	0.013	0.012
Atlantic1	0.011 (0.002)	0.016	0.016	0.014	–	0.011	0.012	0.011	0.012	0.012	0.012
*P. tortonesei*	0.010 (0.003)	0.122	0.127	0.124	0.127	–	0.011	0.012	0.012	0.013	0.013
*P. marmoratus*	0.023 (0.002)	0.152	0.148	0.146	0.151	0.160	–	0.012	0.012	0.013	0.013
*P. minutus*	0.016 (0.003)	0.174	0.172	0.170	0.171	0.182	0.189	–	0.009	0.012	0.012
*P. lozanoi*	0.009 (0.003)	0.176	0.170	0.170	0.171	0.178	0.182	0.105	–	0.013	0.012
*P. canestrinii*	0.001 (0.001)	0.207	0.204	0.206	0.204	0.213	0.208	0.183	0.185	–	0.012
*Knipowitschia*	na	0.181	0.181	0.180	0.179	0.197	0.207	0.154	0.164	0.181	–

a Genetic distance within lineages (S.E. = standard error).

b Genetic distance between lineages (below the diagonal).

c Standard error (above the diagonal).

**Table 2:** Genetic diversity indices for each *Pomatoschistus microps* lineage.

	n^a^	*nh* b	π^c^(sd)	*h* d(sd)	*k* e
Mediterranean	80	42	0.0052 (0.0005)	0.955 (0.014)	5.326
N. European	23	21	0.0092 (0.0007)	0.992 (0.015)	9.561
Atlantic1	86	77	0.0103 (0.0003)	0.995 (0.004)	10.625
Atlantic2	5	4	0.0025 (0.0008)	0.900 (0.025)	2.600

a Number of sequences.

b Number of haplotypes.

c Nucleotide diversity (sd = standard deviation).

d Haplotype diversity (sd = standard deviation).

e Mean number of pairwise differences.

**Figure 3:**
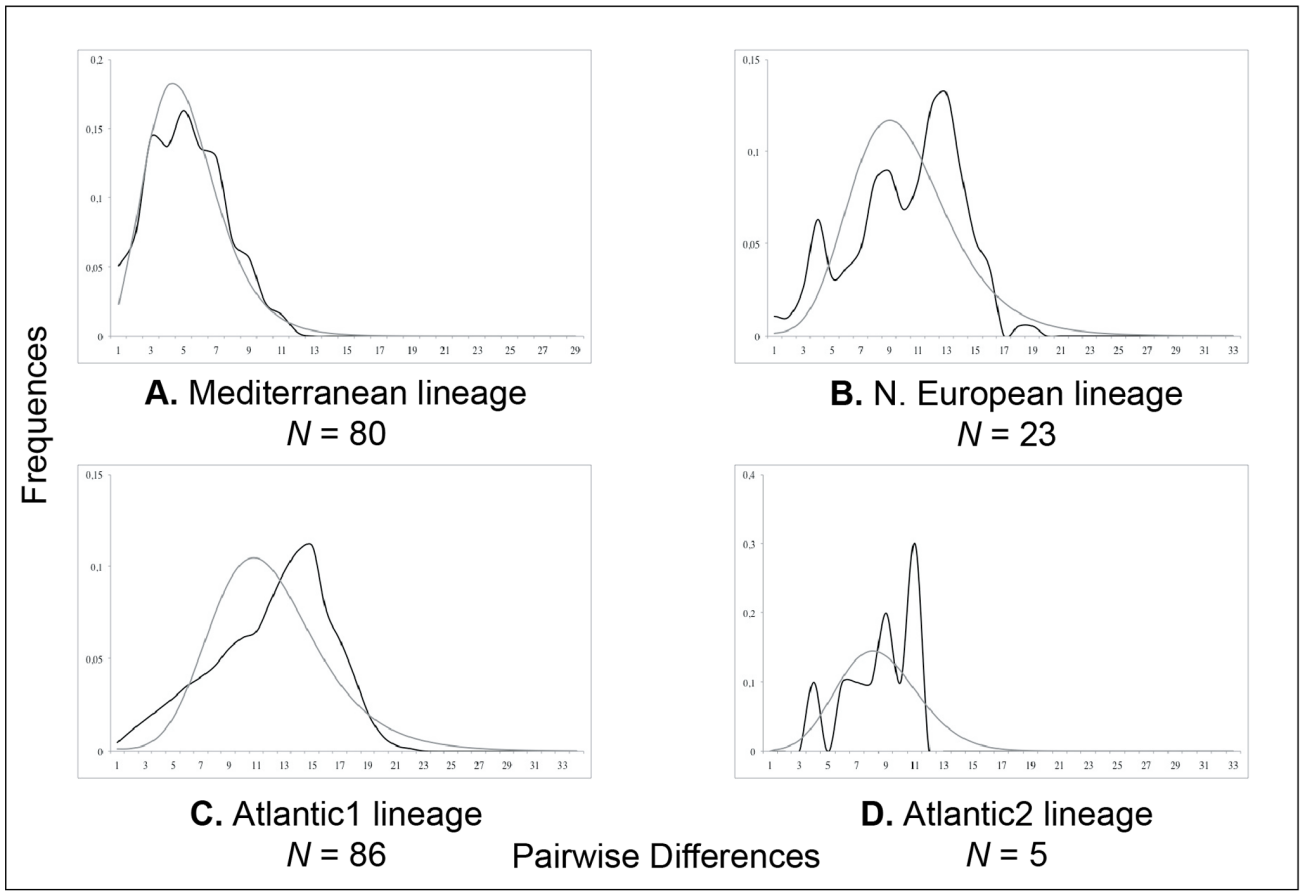
Demographic history of *Pomatoschistus microps* inferred from concatenated control region and cytochrome *b* gene sequences. Observed mismatch distributions (grey line) for each lineage are compared to expected distributions (black line) under a population growth-decline model.

Demographic history of the four *microps* lineages was inferred from three neutrality tests (Tajima’s *D* statistic, Fu’s *F*s and *R*_2_ test) and mismatch distributions (SSD and *r*; Table 3 and Fig. 3). In Fig. 3, unimodal distributions are observed for M and A1 suggesting sudden expansion for these lineages, while results for NE and A2 are more difficult to interpret. Neutrality tests and mismatch distribution indices are not fully in agreement. Differences can be explained by their statistical power to detect population growth [56]. For this reason, a lineage follows the demographic expansion model when results from at least three tests were found significant. Consequently, three *microps* lineages (M, A1 and NE) appear as expanding but not A2.

**Table 3:** Demographic indices for each *Pomatoschistus microps* lineage.

	*F*s^a^	*D* b	*R*_2_ c	SSD^d^	*r* e	Model
Mediterranean	−29.720***^f^	−1.574*	0.052*	0.002	0.009	expansion
N. European	−10.405***	−0.972	0.088	0.006	0.010	expansion
Atlantic1	−87.019***	−1.784*	0.044*	0.032	0.110	expansion
Atlantic2	−0.567	−0.668	0.189	0.002	0.003	stable

a Fu’s *F*s.

b Tajima’s *D.*

c Ramos-Onsins and Rozas’s *R*_2._

d Sum of squared deviations.

e Raggedness index.

f Probability of the test: *** <0.001, ** <0.01, * <0.05.

**Figure 4:**
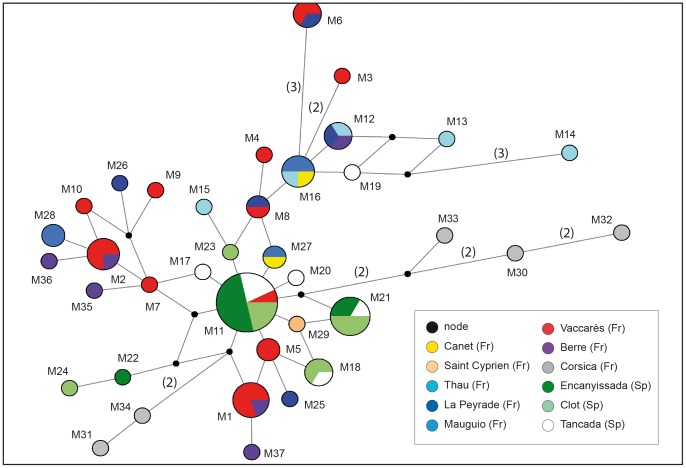
Median-joining network of the concatenated control region and cytochrome *b* gene haplotypes for *Pomatoschistus microps* of the Mediterranean lineage. Haplotypes are indicated by numbers as given in Table S1. Line lengths reflect the number of mutational changes (in brackets when >1) between haplotypes.

**Figure 5:**
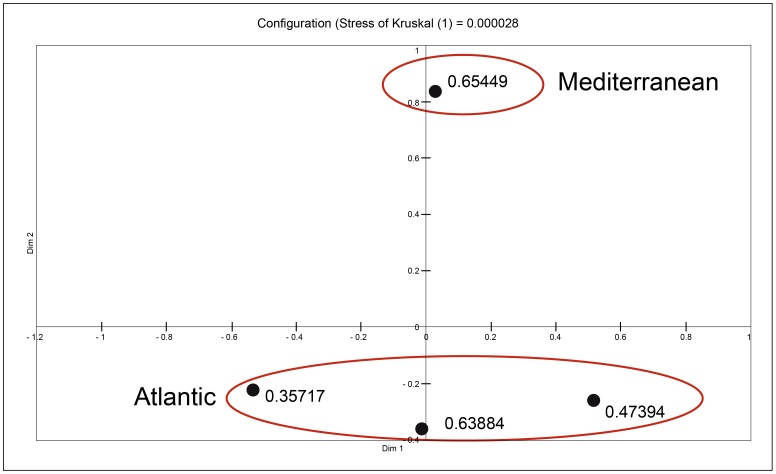
Non-multidimensional scaling plot using pairwise *F*_ST_ values from the four *Pomatoschistus microps* lineages. The Mediterranean group integrates all the common gobies of the Mediterranean lineage, while the Atlantic group includes individuals from the Atlantic lineages (A1, A2 and NE).

Population structure was evaluated with a median-joining network. Because of complex and uninformative reticulated relationships connecting haplotypes of the Atlantic populations, only a sub-network for the Mediterranean lineage haplotypes is presented on Fig. 4. The network obtained with the whole dataset showed the same general population structure as the tree depicted in Fig. 2, with close relationships between the four *microps* lineages. However, if the validity of the four lineages could be questioned from the phylogenetic trees, it is not the case in the network since it displays four well-defined lineages (data not shown). Every lineage is characterized by a large number of haplotypes. One Mediterranean haplotype is dominant for all type of datasets (CR = 24 individuals; cytb = 24 individuals; CR+cytb = 14 individuals). Few haplotypes are shared by several localities (Tables S3 and S4). The analysis of molecular variance revealed significant moderate population structure between the different groups investigated (Φ_ST_ = 0.499 for all *microps* lineages; Φ_ST_ = 0.517 for Mediterranean vs Atlantic lineages; Φ_ST_ = 0.500 for Mediterranean vs Atlantic vs North European lineages; Φ_ST_ = 0.262 for Spanish vs French Mediterranean populations). On the other hand, the two-dimensional space of the NMDS (Fig. 5) based on the *F*_ST_ distance matrix (Kruskal stress value of 0.000028) indicates that the relative distance between Mediterranean and Atlantic lineages reflects their high genetic dissimilarity. The occurrence of some North European (Ireland and Dunkerque) and Mediterranean (Vic lagoon) individuals in the A1 lineage testifies to some gene flow between lineages or to an ancestral polymorphism in the northern Europe or in the Mediterranean.

## Discussion

### Evolutionary History of *Pomatoschistus microps*

The hypothesis of the Strait of Gibraltar, or rather the Almería-Oran Front, acting as a barrier to gene flow between Atlantic and Mediterranean populations of marine plants and animals has been widely investigated in the last decades [11]. Most previous published studies related to the phylogeography of some *Pomatoschistus* species with a North Atlantic-Mediterranean distribution (*P. minutus* and *P. microps*) and based on other molecular markers (283 bp cytb fragment for SSCP analysis for both species; 850 bp cytb fragment for *P. minutus*; 720 bp cytb fragment and EPIC markers for *P. minutus*) [29], [30], [31], [32] underlined a significant differentiation between western Mediterranean populations and those of the Atlantic. In the present study, the phylogeographic pattern of the common goby, *P. microps*, is characterized by a low genetic differentiation of four main evolutionary lineages (M, NE, A1 and A2). A similar pattern was previously observed by [30]. However, these lineages are not reciprocally monophyletic (weak BP or PP values in Fig. 2 or for the tree obtained with the dataset of [31]). Low nucleotide diversity (Table 2), polytomic topologies (Fig. 2) and demographic analyses (Table 3 and Fig. 3) provide evidence of population expansion for the M, NE and A1 lineages. The weak number of individuals included in A2 does not allow to test the hypothesis of demographic expansion. In most studies, a low genetic diversity associated with demographic indices indicating sudden expansion are interpreted as past bottleneck events followed by post-glacial recolonization [11], [58], [63], [64], [65], [66], [67]. Here, the molecular dating suggests a young origin for *P. microps* and its lineages (between 77,000 and 54,000 yr; Weichselian glacial period). This information combined with the tree topologies (basal polytomy and shallow branching pattern), phylogeographic (moderate to high population structure), and demographic (expansion) analyses as well as high haplotype diversity rather underline a recent and rapid population divergence during the last glacial [68], [69], [70], [71]. This period was characterized by a climatic instability with 24 interstadials (IS), *i.e*. warm period during a glacial period [72], [73]. If our molecular divergence times are compared with δ^18^O record [72], it seems that each divergence event of *P. microps* occurred between two glacial interstadials. First, a population of the *P. microps* ancestor could have been trapped in Atlantic between IS 20 and IS 21. Because of the basal position of Portuguese and French Atlantic individuals in the *P. microps* clade as well as the occurrence of numerous Atlantic individuals in all lineages, the Mediterranean origin of *P. microps* previously proposed by [30] is not here supported. From the first Atlantic divergent population, *P. microps* seems to have colonized northern Europe during the next interstadials. Because of colder and drier climatic conditions, some populations became extinct, while others survived in some glacial refugia. A glacial refugium was previously proposed for *P. microps* in the southern North Sea as well as for *P. minutus* during the Weichselian period [29], [30]. The composition of the NE lineage (mainly individuals from northern France, Ireland and Scotland) and our molecular dating confirm this idea of a glacial refugium located in northern Europe. A second glacial refugium has been identified between the Bay of Biscay and the Iberian Peninsula [29], [30], [31], [32]. The occurrence of the A1 lineage clustering mainly individuals from Atlantic Portuguese and French localities strengthens this idea. Because of the occurrence of some individuals from the same Atlantic Portuguese and French localities in the NE lineage or as the first offshoot of the M lineage, these populations seem also to have contributed to the recolonization of northern Europe and to the colonization of the Mediterranean Sea.

Most studies previously published on *P. microps* [30] and on *P. minutus* [29], [31], [32] display similar phylogeographic pattern (biogeographic separation of Atlantic and Mediterranean populations) and population structure (restricted gene flow). Similar life-history traits could explain these concordant evolutionary histories. However, the present study suggests another evolutionary history, probably because new sampling strategies have been applied in the present study. Gysels *et al*. [30] analysed discontinuous and unbalanced samples: South European populations (and especially around the Gibraltar Strait) being under-represented compared to North European populations. As far as, the Atlantic-Mediterranean transition seems a crucial point for the evolutionary history of marine plants and animals, this area was here over-sampled with several Portuguese (8), Spanish (6) and French Mediterranean (12) localities. In this latter context, evolutionary histories of *P. microps* and *P. minutus* appear different. Phylogeographic studies have sometimes disclosed discordant results for closely related species with similar life-history traits. Several hypotheses are proposed to explain these discrepancies, for instance: loss of some ancestral clades during less suitable periods and re-occupation of the vacant niche by other clades in expansion; different responses to the presence of oceanographic barriers and/or fluctuations in effective population sizes [11], [74], [75]. The observed difference in the degree of genetic differentiation between *P. microps* and *P. minutus* could rather lie in the time passed since the appearance of these species or in different reproduction cycles. *P. microps* seems much younger (77,000 yr) than *P. minutus* (300,000 yr) [32]. Sufficient time did not probably pass yet for *P. microps* to reach the same degree of differentiation than *P. minutus*.

### Colonisation of the Mediterranean Sea by *Pomatoschistus microps*

Although Quaternary sea-level lowering (up to −105/−115 m during the Last Glacial Maximum along the French Mediterranean coasts) [13] did not interrupt the movement of organisms through the Strait of Gibraltar, they limited it because of restricted water exchanges between the Atlantic and the MS that lead to habitat fragmentation [74]. This sea-level lowering associated with colder and drier conditions during glacial periods also induced sea-surface salinity increase and temperature decrease [12]. Among the various ecological parameters influenced by weather conditions, salinity and temperature are the most important for fish adaptation. Experiments on osmoregulation showed that *P. microps* has a wide salinity tolerance, and notably a larger range of tolerance than the closely related species *P. marmoratus* [76]. The common goby seems particularly tolerant to low salinities down to freshwater conditions. *P. microps* is besides the dominant or even exclusively present sedentary goby in brackish lagoons of the western Mediterranean with low or unstable (0–40) salinity conditions as in the Canet, Mauguio and Biguglia lagoons [37], [76]. This physiological capability could have allowed this goby to survive to the salinity increase in the Mediterranean Sea during glacial periods, and to resist the glacial meltwater supply from the river runoff and Atlantic inflow during interstadial deglaciations [12], [77]. Several life-history traits (life cycle, growth, migration, reproduction, size, life time) [36] of the common goby appear different in the Atlantic and Mediterranean. This shift in life-history traits should have accompanied the settlement of the Mediterranean lineage in new environmental conditions during the Late Weichselian, and the divergence of the Mediterranean lineage from the Atlantic ones.

Due to its life cycle entirely accomplished inside lagoons, Gysels *et al*. [30] expected a high genetic structure of *P. microps* in Mediterranean lagoons. Indeed, the samples from the GDL (Pérols and Vaccarès) analysed with allozymes and SSCP mtDNA were grouped together. No genetic structure was highlighted here either neither in the western Mediterranean nor in the GDL (Fig. 4 and Φ_ST_ results), although 18 Mediterranean localities were investigated. One haplotype is dominant (M11) and shared by individuals from the complex of Vaccarès lagoons (GDL, France) and mostly from the Ebro Delta (Spain). The Mediterranean haplotype network exhibits a star-like pattern with few mutation events (1 to 3) and organized around this dominant and presumably ancestral haplotype for the Mediterranean lineage. Twenty-five out of the 37 endemic Mediterranean haplotypes are private haplotype, the majority being the most divergent haplotypes compared to the dominant one. Few haplotypes are thus shared between lagoons, as also observed by [78]. This network pattern is usually regarded as indicative of populations that have expanded their range recently from a small founder number [79]. This is concordant with our molecular dating for the Mediterranean lineage (54,000 yr; Fig. 2) and our hypothesis about the Atlantic populations at the origin of the Mediterranean ones. On the other hand, it seems difficult to directly link the results obtained for the Mediterranean lineage with the recent formation of the Mediterranean lagoons (for instance, final closure of some GDL lagoons during the last millennium) [16]. On the basis of RFLP from mtDNA (CR+cytb), Berrebi *et al*. [78] did not observed common shared haplotypes between the GDL lagoons. They deduced that no significant exchange of migrants occurred between lagoons for centuries. However, some shared haplotypes are found between more or less distant localities in the present study. A similar structure has been found for *P. minutus* [32], but *P. minutus* is a migratory species leaving lagoons to reproduce in the open sea [36]. For the Mediterranean sedentary *P. microps*, we can hypothesize that shared haplotypes, including the dominant haplotype, dispersed in the western Mediterranean basin before the lagoon formation, while most private haplotypes, evidencing a recent isolation, could have diverged in lagoons after their closure.

## Conclusions

Molecular dating estimates (young origin for *P. microps*) combined with tree topologies (polytomy), phylogeographic (no significant genetic break between Atlantic and Mediterranean populations) and demographic (expansion) analyses as well as high haplotype diversity underline a recent and rapid population divergence during the last glacial. These results are in contradiction with previous published studies based on other molecular markers (cytb SSCP and allozymes). Present investigations on the population structure with AMOVA and NMDS analyses (*e.g*. indirect estimates of gene flow) suggest however limited genetic exchanges between Atlantic and Mediterranean lineages. From Portuguese ancestral populations and glacial refugia, the common goby could have colonized first northern Europe and then the Mediterranean Sea.

*Pomatoschistus microps* is tolerant to low salinity conditions. For this reason, Quaternary salinity variations in the Mediterranean Sea (salinity increase during glacial periods interrupted by interstadial periods with river freshwater runoff) could be at the origin of its Mediterranean settlement. Shared haplotypes could have dispersed in the western Mediterranean basin before the lagoon formation, while most private haplotypes, evidencing a recent isolation, diverged in lagoons after their closure.

According to Paternello *et al*. [11], three phylogeographic scenarios of the Atlantic-Mediterranean populations are possible: (1) lack of population structure because of high gene flow between Atlantic and Mediterranean populations; (2) high genetic differentiation (historic separation, recent isolation, distinct population dynamics) between Atlantic and Mediterranean populations with the Almería-Oran Front as a phylogeographic break; (3) eastern Mediterranean populations forming a distinct clade from the one clustering Atlantic and western Mediterranean populations. However, from the present study, a fourth scenario can be proposed. A recent and rapid divergence with no significant genetic differentiation but population structure suggests that differentiation is an ongoing process at the Atlantic-Mediterranean scale as well as in the Mediterranean lagoons.

## Supporting Information

Figure S1 **Maximum-likelihood tree reconstructed from control region sequences from** ***Pomatoschistus microps*** **.** Individual labels are detailed in Table S1. Numbers at nodes are for ML bootstrap percentages (≥50%). *P. microps* lineages are indicated on the right.
(PDF)

Figure S2 **Bayesian tree reconstructed from control region sequences from** ***Pomatoschistus microps*** **.** Individual labels are detailed in Table S1. Numbers at nodes are for BI posterior probabilities (≥0.80). *P. microps* lineages are indicated on the right.
(PDF)

Figure S3 **Maximum-likelihood tree reconstructed from cytochrome** ***b*** **gene sequences from** ***Pomatoschistus microps*** **.** Individual labels are detailed in Table S1. Numbers at nodes are for ML bootstrap percentages (≥50%) and BI posterior probabilities (≥0.80). *P. microps* lineages are indicated on the right.
(PDF)

Figure S4 **Bayesian tree reconstructed from concatenated control region and cytochrome** ***b*** **gene sequences from** ***Pomatoschistus microps*** **.** Individual labels are detailed in Table S1. Numbers at nodes are for BI posterior probabilities (≥0.80). *P. microps* lineages are indicated on the right.
(PDF)

Table S1 **Detailed list of labels, localities and references or tissue providers of** ***Pomatoschistus microps*** **samples and outgroup species.** Accession numbers for original and GenBank sequences as well as control region and/or cytochrome *b* gene haplotypes are also listed. G vouchers are for samples stored at ISEM (Montpellier, France), while Pm vouchers are from the Centro de Oceanografia (Lisbon, Portugal).
(PDF)

Table S2 **Alternative topologies non-significantly different (5% confidence level) including the four lineages of** ***Pomatoschistus microps.***
(PDF)

Table S3 **Distribution and number of concatenated control region and cytochrome** ***b*** **gene haplotypes per locality (Sc, Scotland; Ire, Ireland).** The haplotype number is in the first column, while the locality number (Fig. 1 and Table S1) is in the first row.
(PDF)

Table S4 **Distribution and number of the control region (light grey) and cytochrome** ***b*** **gene (dark grey) haplotypes per locality (Sc, Scotland; Ire, Ireland).** The haplotype number is in the first column, while the locality number (Fig. 1 and Table S1) is in the first (CR) and second (cytb) rows.
(XLS)
