# Supplementary figures and images for: New Light on the Evolutionary History of the Common Goby (Pomatoschistus microps) with an Emphasis on Colonization Processes in the Mediterranean Sea
Source: PLoS One. 2014 Mar 19;9(3):e91576. doi: 10.1371/journal.pone.0091576 (PMC3960122; doi:10.1371/journal.pone.0091576)

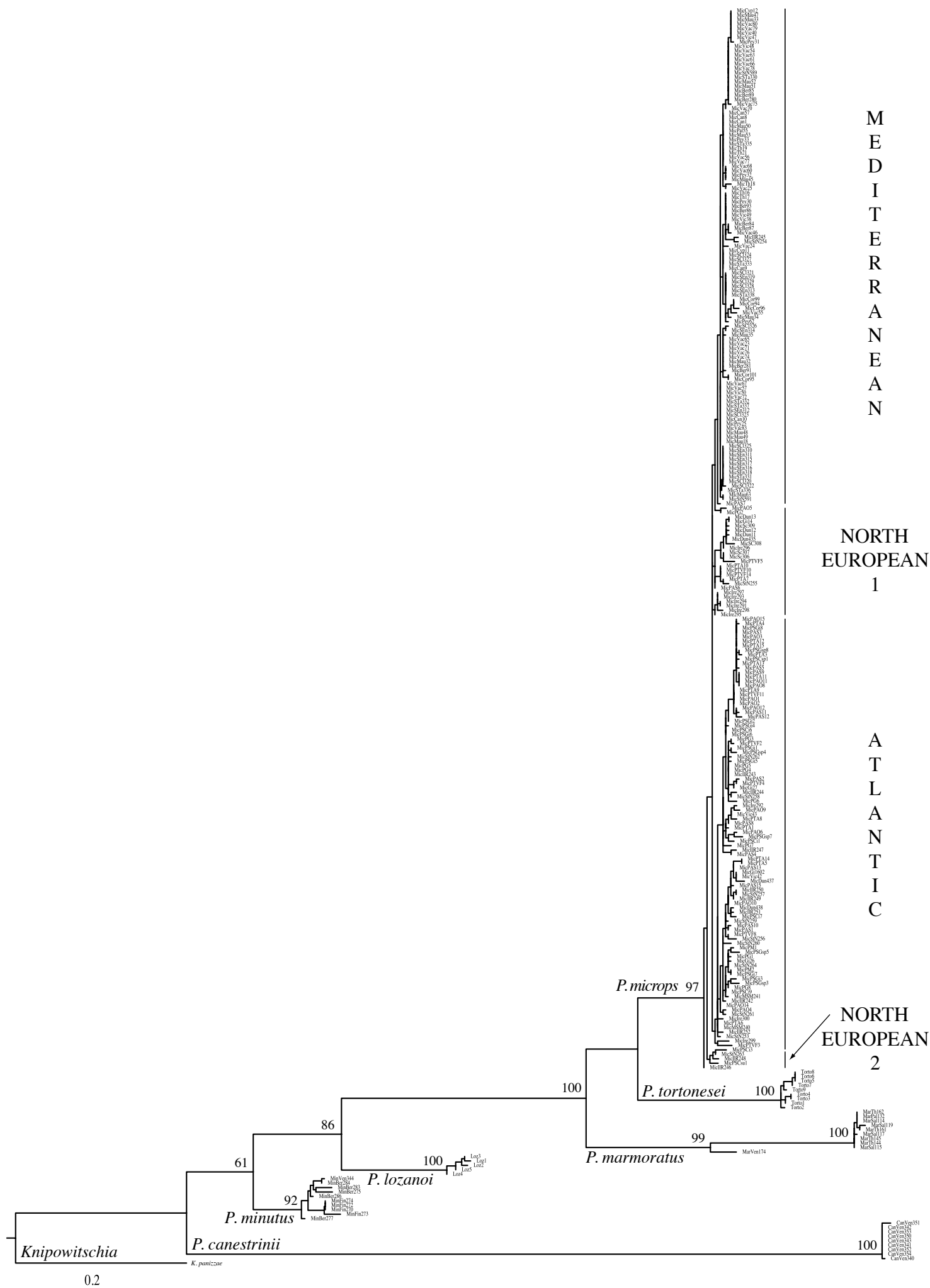

Supplement: Figure S1 — Maximum-likelihood tree reconstructed from control region sequences from Pomatoschistus microps . Individual labels are detailed in Table S1. Numbers at nodes are for ML bootstrap percentages (≥50%). P. microps lineages are indicated on the right. (PDF) [file pone.0091576.s001.pdf]

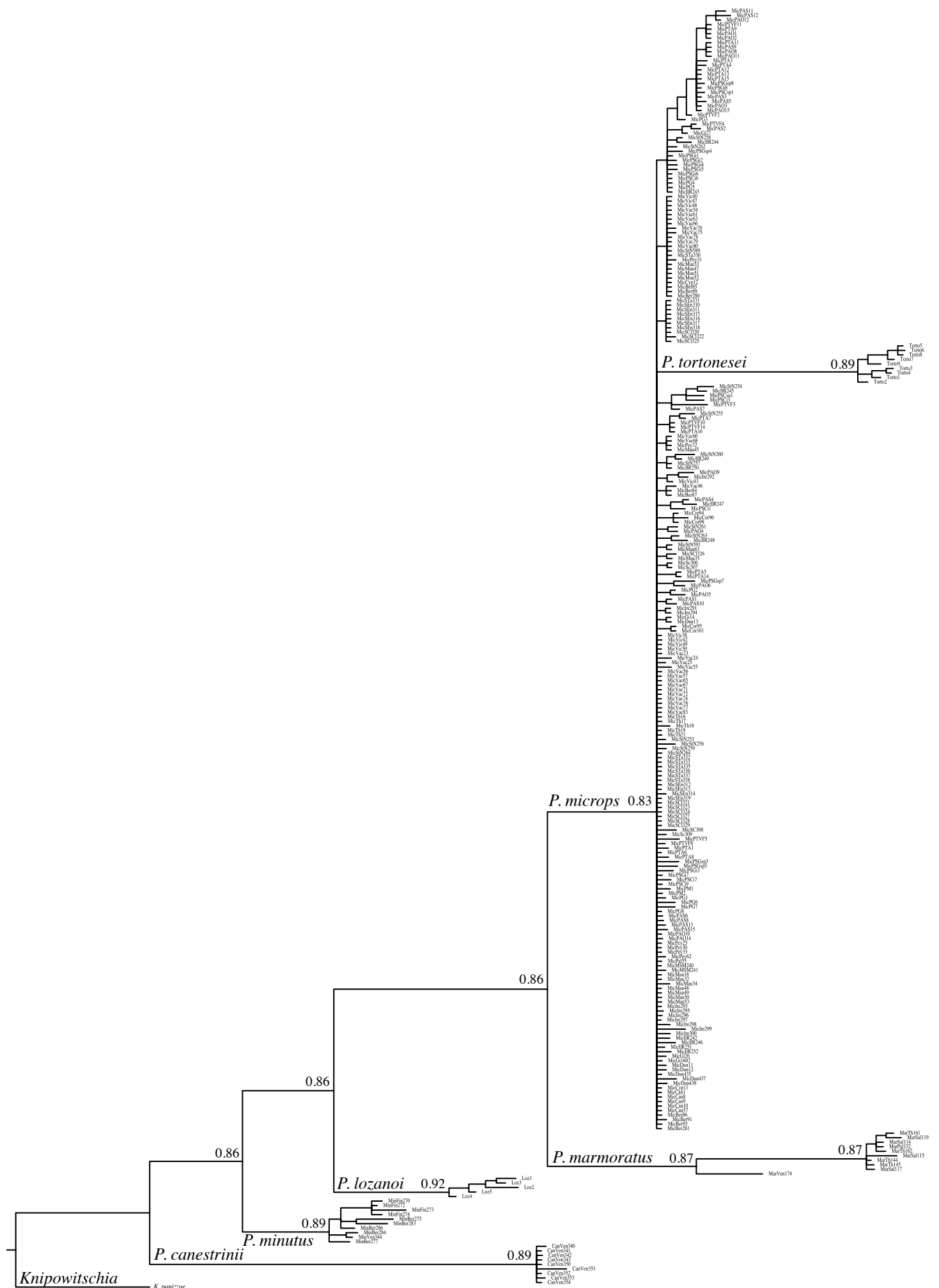

0.6

Supplement: Figure S2 — Bayesian tree reconstructed from control region sequences from Pomatoschistus microps . Individual labels are detailed in Table S1. Numbers at nodes are for BI posterior probabilities (≥0.80). P. microps lineages are indicated on the right. (PDF) [file pone.0091576.s002.pdf]

NORTH  
EUROPEAN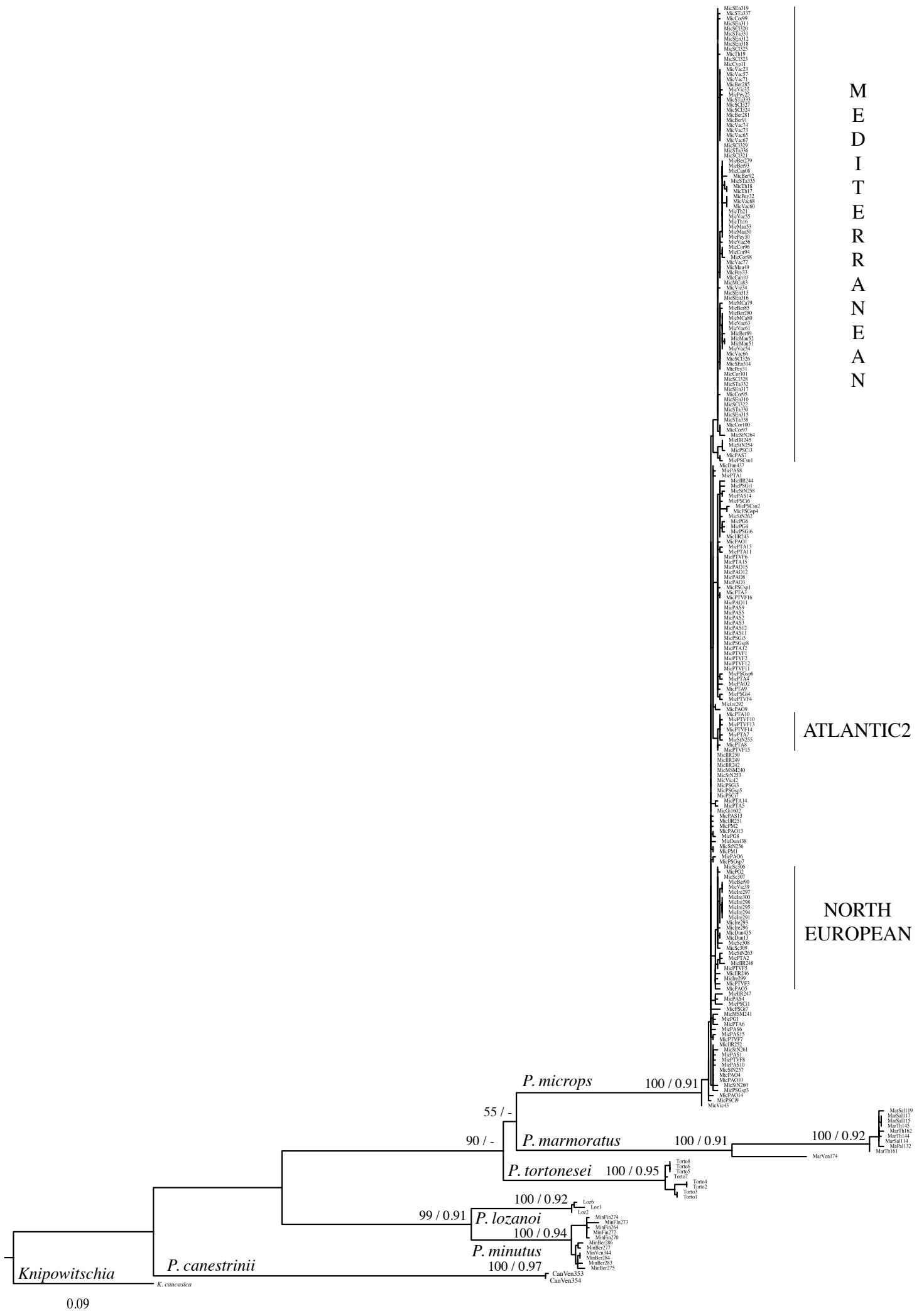

Supplement: Figure S3 — Maximum-likelihood tree reconstructed from cytochrome b gene sequences from Pomatoschistus microps . Individual labels are detailed in Table S1. Numbers at nodes are for ML bootstrap percentages (≥50%) and BI posterior probabilities (≥0.80). P. microps lineages are indicated on the right. (PDF) [file pone.0091576.s003.pdf]

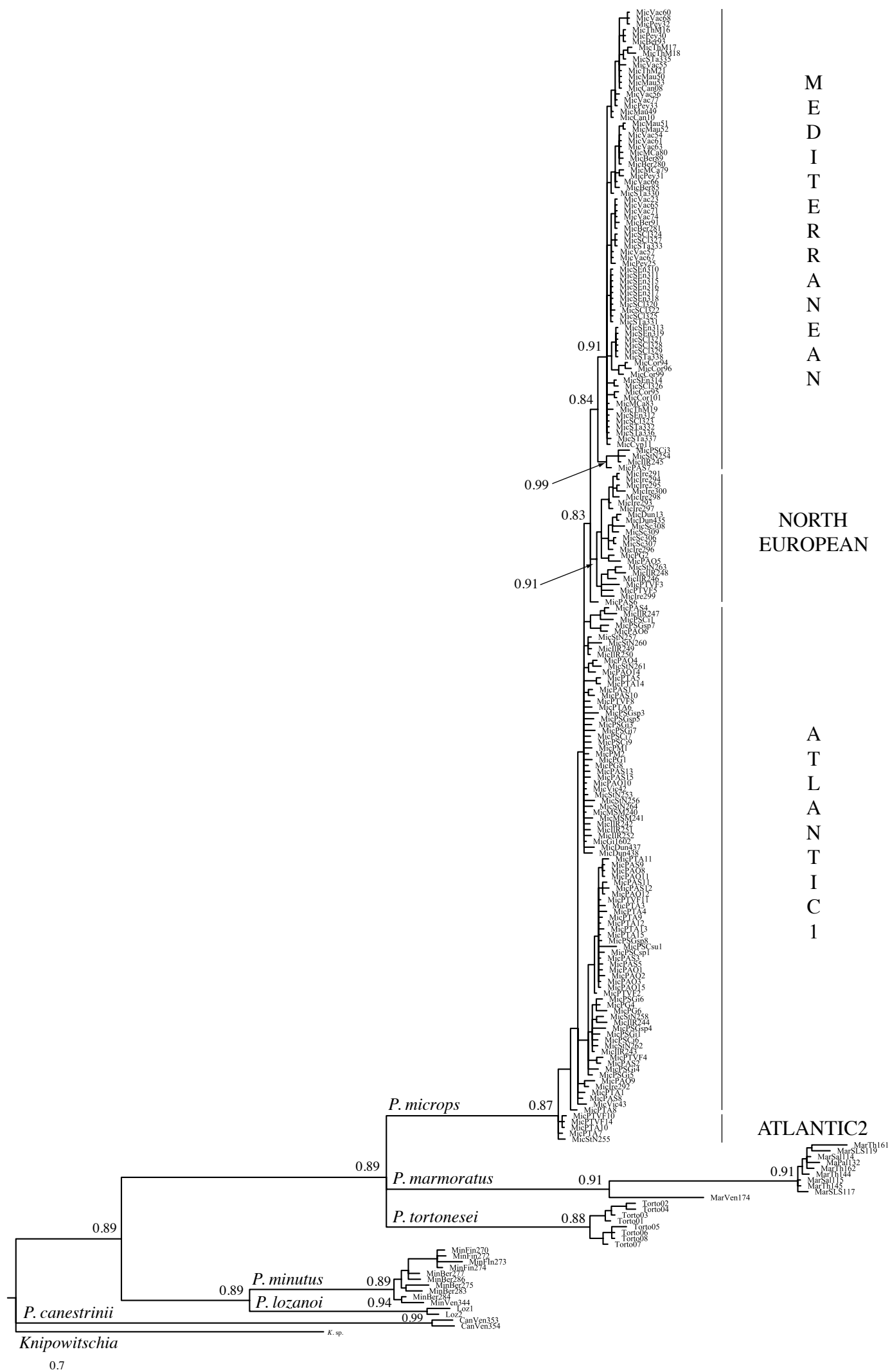

Supplement: Figure S4 — Bayesian tree reconstructed from concatenated control region and cytochrome b gene sequences from Pomatoschistus microps . Individual labels are detailed in Table S1. Numbers at nodes are for BI posterior probabilities (≥0.80). P. microps lineages are indicated on the right. (PDF) [file pone.0091576.s004.pdf]
