# Supplementary material for: New Light on the Evolutionary History of the Common Goby (Pomatoschistus microps) with an Emphasis on Colonization Processes in the Mediterranean Sea
Source: PLoS One. 2014 Mar 19;9(3):e91576. doi: 10.1371/journal.pone.0091576 (PMC3960122; doi:10.1371/journal.pone.0091576)
Supplement: Table S1 — Detailed list of labels, localities and references or tissue providers of Pomatoschistus microps samples and outgroup species. Accession numbers for original and GenBank sequences as well as control region and/or cytochrome b gene haplotypes are also listed. G vouchers are for samples stored at ISEM (Montpellier, France), while Pm vouchers are from the Centro de Oceanografia (Lisbon, Portugal). (PDF) [file pone.0091576.s005.pdf]

Taxa	Country	Geographic area	Shore area / Lagoon	Locality	Voucher	Label	Control Region		Cytochrome b		Haplotype	Tissue providers or bibliographic references[a]
							Accession number	Haplotype Hap_{CR}	Accession number	Haplotype Hap_{CB}	CR+CB Hap_{Comb}	
	Scotland, UK	Firth of Clyde	1. Kames Bay	Great Cumbrae Island	G3233	MicSc306	HF969335	34	HF969614	102	NE2	M. Hardman
					G3234	MicSc307	HF969336	34	HF969615	102	NE2	M. Hardman
					G3235	MicSc308	HF969337	35	HF969616	103	NE3	M. Hardman
					G3236	MicSc309	HF969338	36	HF969617	104	NE4	M. Hardman
	Ireland	Atlantic	Ireland Sea	2. Wexford	G3202	MicIre291	HF969339	101	HF969618	89	NE10	S. Mariani
					G3203	MicIre292	HF969340	102	HF969619	90	A1_67	S. Mariani
					G3204	MicIre293	HF969341	103	HF969620	91	NE11	S. Mariani
					G3205	MicIre294	HF969342	101	HF969621	89	NE10	S. Mariani
					G3206	MicIre295	HF969343	104	HF969622	89	NE12	S. Mariani
					G3207	MicIre296	HF969344	105	HF969623	92	NE13	S. Mariani
					G3208	MicIre297	HF969345	103	HF969624	74	NE14	S. Mariani
					G3209	MicIre298	HF969346	106	HF969625	89	NE15	S. Mariani
					G3210	MicIre299	HF969347	107	HF969626	93	NE16	S. Mariani
					G3211	MicIre300	HF969348	108	HF969627	89	NE17	S. Mariani
	France	The Channel		3. Dunkerque	G0436	MicDun11	HF969349	121				ISEM
					G0439	MicDun12	HF969350	122				ISEM
					G0445	MicDun13	HF969351	118	HF969628	105	NE20	ISEM
					G0435	MicDun435	HF969352	123	HF969629	105	NE21	ISEM
					G0437	MicDun437	HF969353	124	HF969630	106	A1_76	ISEM
					G0438	MicDun438	HF969354	125	HF969631	107	A1_77	ISEM
			Mont Saint Michel Bay	4. Cherrueix	G3124	MicMSM240	HF969355	48	HF969632	25	A1_65	A. Carpentier & E. Feunteun
					G3125	MicMSM241	HF969356	98	HF969633	86	A1_66	A. Carpentier & E. Feunteun
		Atlantic	Loire Mouth	5. Saint Nazaire	G3137	MicStN253	HF969357	17	HF969634	25	A1_3	A. Carpentier & E. Feunteun
					G3138	MicStN254	HF969358	18	HF969635	78	M38	A. Carpentier & E. Feunteun
					G3139	MicStN255	HF969359	19	HF969636	79	A2_1	A. Carpentier & E. Feunteun
					G3140	MicStN256	HF969360	20	HF969637	39	A1_4	A. Carpentier & E. Feunteun
					G3141	MicStN257	HF969361	21	HF969638	57	A1_5	A. Carpentier & E. Feunteun
					G3142	MicStN258	HF969362	22	HF969639	80	A1_6	A. Carpentier & E. Feunteun
					G3143	MicStN259	HF969363	23				A. Carpentier & E. Feunteun
					G3144	MicStN260	HF969364	24	HF969640	81	A1_7	A. Carpentier & E. Feunteun
					G3145	MicStN261	HF969365	25	HF969641	82	A1_8	A. Carpentier & E. Feunteun
					G3146	MicStN262	HF969366	26	HF969642	83	A1_9	A. Carpentier & E. Feunteun
					G3147	MicStN263	HF969367	27	HF969643	84	NE1	A. Carpentier & E. Feunteun
					G3148	MicStN264	HF969368	28	HF969644	85	A1_10	A. Carpentier & E. Feunteun
					G0589	MicStN589	HF969369	5				ISEM
					G0591	MicStN591	HF969370	29				ISEM
			Ile de Ré Fiers d'Ars Bay	6. Lilleau des Niges	G3126	MicIlR242	HF969371	109	HF969645	25	A1_68	A. Carpentier & E. Feunteun
					G3127	MicIlR243	HF969372	75	HF969646	94	A1_69	A. Carpentier & E. Feunteun
					G3128	MicIlR244	HF969373	110	HF969647	95	A1_70	A. Carpentier & E. Feunteun
					G3129	MicIlR245	HF969374	111	HF969648	78	M42	A. Carpentier & E. Feunteun
					G3130	MicIlR246	HF969375	112	HF969649	96	NE18	A. Carpentier & E. Feunteun
					G3131	MicIlR247	HF969376	113	HF969650	97	A1_71	A. Carpentier & E. Feunteun
					G3132	MicIlR248	HF969377	114	HF969651	98	NE19	A. Carpentier & E. Feunteun
					G3133	MicIlR249	HF969378	115	HF969652	25	A1_72	A. Carpentier & E. Feunteun
					G3134	MicIlR250	HF969379	21	HF969653	25	A1_73	A. Carpentier & E. Feunteun
					G3135	MicIlR251	HF969380	116	HF969654	99	A1_74	A. Carpentier & E. Feunteun
					G3136	MicIlR252	HF969381	117	HF969655	57	A1_75	A. Carpentier & E. Feunteun
			Garonne Mouth	7. Gironde estuary	G1607	MicGi14	HF969382	118				ISEM
					G1609	MicGi26	HF969383	119				ISEM
					G1604	MicGi27	HF969384	120				ISEM
					G1602	MicGi1602	HF969385	3	HF969656	25	A1_1	ISEM
		Gulf of Lion	8. Canet-Saint Cyprien Lagoon		G2465	MicCan1	HF969386	12				ISEM
					G2472	MicCan8	HF969387	12	HF969657	65	M16	ISEM
					G2473	MicCan9	HF969388	30				ISEM
					G2475	MicCan10	HF969389	6	HF969658	69	M27	ISEM
					G2422	MicCan57	HF969390	12				ISEM
					G2718	MicCyp11	HF969391	30	HF969659	71	M29	ISEM
					G2738	MicCyp12	HF969392	2				ISEM
			9. La Palme Lagoon		G2367	MicPal55	HF969393	12				ISEM
			Thau Lagoon	10. Marseillan	G2801	MicTh16	HF969394	1	HF969660	65	M12	ISEM
					G2802	MicTh17	HF969395	1	HF969661	76	M13	ISEM
					G2803	MicTh18	HF969396	16	HF969662	76	M14	ISEM
					G2804	MicTh19	HF969397	12	HF969663	76	M15	ISEM
					G2806	MicTh21	HF969398	12	HF969664	65	M16	ISEM
			11. La Peyrade Lagoon		G2502	MicPey25	HF969399	6	HF969665	88	M25	ISEM
					G2508	MicPey30	HF969400	1	HF969666	65	M12	ISEM
					G2509	MicPey31	HF969401	96	HF969667	68	M26	ISEM
					G2510	MicPey32	HF969402	13	HF969668	67	M6	ISEM
					G2511	MicPey33	HF969403	12	HF969669	69	M8	ISEM
					G2993	MicPey62	HF969404	97				ISEM
			12. Vic Lagoon		G2003	MicVic34			HF969670	72		ISEM
					G2004	MicVic35			HF969671	73		ISEM
					G2775	MicVic38	HF969405	1				ISEM
					G2777	MicVic39			HF969672	74		ISEM
					G2778	MicVic40	HF969406	2				ISEM
					G2780	MicVic42	HF969407	3	HF969673	25	A1_1	ISEM
					G2781	MicVic43	HF969408	4	HF969674	75	A1_2	ISEM
					G2005	MicVic47	HF969409	2				ISEM
					G2006	MicVic48	HF969410	5				ISEM
					G2774	MicVic49	HF969411	1				ISEM
					G2776	MicVic50	HF969412	6				ISEM
			Mauguio Lagoon	13. Mauguio	G1194	MicMau18	HF969413	6				ISEM
					G1190	MicMau32	HF969414	7				ISEM
					G1217	MicMau33	HF969415	2				ISEM
					G1503	MicMau34	HF969416	99				ISEM
					G1506	MicMau35	HF969417	100				ISEM
					G1202	MicMau45	HF969418	13				ISEM
					G1204	MicMau47	HF969419	2				ISEM
					G1205	MicMau48	HF969420	6				ISEM
					G1191	MicMau63	HF969421	29				ISEM
					G2623	MicMau49	HF969422	6	HF969675	69	M27	ISEM
					G2624	MicMau50	HF969423	12	HF969676	65	M16	ISEM
					G2625	MicMau51	HF969424	5	HF969677	87	M28	ISEM
					G2626	MicMau52	HF969425	5	HF969678	87	M28	ISEM
					G2627	MicMau53	HF969426	12	HF969679	65	M16	ISEM
			Complex of Vaccarés Lagoons	14. Malagroy Lagoon	G2668	MicVac23	HF969427	7	HF969680	63	M1	ISEM
					G2671	MicVac24	HF969428	8				ISEM
					G2675	MicVac25	HF969429	9				ISEM
					G0366	MicVac60	HF969430	13	HF969681	67	M6	ISEM
					G0367	MicVac61	HF969431	5	HF969682	64	M2	ISEM
					G0369	MicVac63	HF969432	5	HF969683	64	M2	ISEM
					G2660	MicVac74	HF969433	7	HF969684	63	M1	ISEM
					G2661	MicVac75	HF969434	15				ISEM
					G2662	MicVac76	HF969435	7				ISEM
					G2663	MicVac77	HF969436	12	HF969685	69	M8	ISEM
					G2664	MicVac78	HF969437	5				ISEM
					G1256	MicVac46	HF969438	10				ISEM
					G1223	MicVac79	HF969439	2	HF969686	70	M9	ISEM
					G1224	MicVac80	HF969440	2	HF969687	64	M10	ISEM
					G1227	MicVac83	HF969441	6	HF969688	71	M11	ISEM
				15. Mornés	G1071	MicVac65	HF969613	7	HF969689	63	M1	ISEM
					G1072	MicVac66	HF969443	5	HF969690	68	M7	ISEM
					G1073	MicVac67	HF969444	6	HF969691	63	M5	ISEM
					G1074	MicVac68	HF969445	13	HF969692	67	M6	ISEM
				16. Fiélouze	G0264	MicVac54	HF969446	5	HF969693	64	M2	ISEM
					G0265	MicVac55	HF969447	11	HF969694	65	M3	ISEM
					G0256	MicVac56	HF969448	12	HF969695	66	M4	ISEM
					G0259	MicVac57	HF969449	6	HF969696	63	M5	ISEM
				17. Capelière	G0829	MicVac70	HF969450	14				ISEM
					G0830	MicVac71	HF969451	7	HF969697	63	M1	ISEM
					G0831	MicVac72	HF969452	6				ISEM
					G0832	MicVac73			HF969698	63		ISEM
			18. Berre Lagoon		G2611	MicBer84	HF969453	129				ISEM
					G2612	MicBer85	HF969454	5	HF969699	113	M35	ISEM
					G2613	MicBer86	HF969455	1				ISEM
					G2614	MicBer87	HF969456	129				ISEM
					G2617	MicBer89	HF969457	5	HF969700	114	M36	ISEM
					G2618	MicBer90			HF969701	74		ISEM
					G2620	MicBer91	HF969458	130	HF969702	63	M37	ISEM
					G2621	MicBer92			HF969703	115		ISEM
					G2622	MicBer93	HF969459	1	HF969704	65	M12	ISEM
					G3172	MicBer279			HF969705	65		L. Brosse
					G3173	MicBer280	HF969460	5	HF969706	64	M2	L. Brosse
					G3174	MicBer281	HF969461	7	HF969707	63	M1	L. Brosse
					G3187	MicBer285			HF969708	63		L. Brosse
					G0042	MicCor94	HF969462	126	HF969709	108	M30	ISEM
					G0043	MicCor95	HF969463	107	HF969710	109	M31	ISEM
					G0045	MicCor96	HF969464	128	HF969711	108	M32	ISEM

	Corsica		19. Biguglia Lagoon		G0081	MicCor97			HF969712	110		ISEM
					G0047	MicCor98			HF969713	111		ISEM
					G0087	MicCor99	HF969465	126	HF969714	112	M33	ISEM
					G0049	MicCor100			HF969715	110		ISEM
					G0050	MicCor101	HF969466	127	HF969716	71	M34	ISEM
	Spain	Ebro Delta	Encanyissada Lagoon	20. P1	G3237	MicSEn310	HF969467	6	HF969717	71	M11	S. Rodriguez
					G3238	MicSEn311	HF969468	6	HF969718	71	M11	S. Rodriguez
					G3239	MicSEn312	HF969469	6	HF969719	71	M11	S. Rodriguez
					G3240	MicSEn313	HF969470	31	HF969720	71	M21	S. Rodriguez
					G3241	MicSEn314	HF969471	32	HF969721	68	M22	S. Rodriguez
				21. P3	G3242	MicSEn315	HF969472	6	HF969722	71	M11	S. Rodriguez
					G3243	MicSEn316	HF969473	6	HF969723	71	M11	S. Rodriguez
					G3244	MicSEn317	HF969474	6	HF969724	71	M11	S. Rodriguez
					G3245	MicSEn318	HF969475	6	HF969725	71	M11	S. Rodriguez
					G3246	MicSEn319	HF969476	31	HF969726	71	M21	S. Rodriguez
			Clot Lagoon	22. P2	G3247	MicSCl320	HF969477	6	HF969727	71	M11	S. Rodriguez
					G3248	MicSCl321	HF969478	31	HF969728	71	M19	S. Rodriguez
					G3249	MicSCl322	HF969479	12	HF969729	71	M23	S. Rodriguez
					G3250	MicSCl323	HF969480	6	HF969730	71	M11	S. Rodriguez
					G3251	MicSCl324	HF969481	30	HF969731	63	M18	S. Rodriguez
				23. P3	G3252	MicSCl325	HF969482	6	HF969732	71	M11	S. Rodriguez
					G3253	MicSCl326	HF969483	33	HF969733	68	M24	S. Rodriguez
					G3254	MicSCl327	HF969484	30	HF969734	63	M18	S. Rodriguez
					G3255	MicSCl328	HF969485	31	HF969735	71	M21	S. Rodriguez
					G3256	MicSCl329	HF969486	31	HF969736	71	M21	S. Rodriguez
			Tancada Lagoon	24. P3	G3257	MicSTa330	HF969487	5	HF969737	71	M17	S. Rodriguez
					G3258	MicSTa331	HF969488	6	HF969738	71	M11	S. Rodriguez
					G3259	MicSTa332	HF969489	6	HF969739	71	M11	S. Rodriguez
					G3260	MicSTa333	HF969490	30	HF969740	63	M18	S. Rodriguez
				25. P5	G3262	MicSTa335	HF969491	12	HF969741	100	M19	S. Rodriguez
					G3263	MicSTa336	HF969492	6	HF969742	71	M11	S. Rodriguez
					G3264	MicSTa337	HF969493	6	HF969743	101	M20	S. Rodriguez
					G3265	MicSTa338	HF969494	31	HF969744	71	M21	S. Rodriguez
	Portugal	Atlantic	Aveiro River	26. Ovar	PmAvOv5091i	MicPAO1	HF969495	43	HF969745	55	A1_58	J. F. Marques
					PmAvOv5092i	MicPAO2	HF969496	43	HF969746	56	A1_59	J. F. Marques
					PmAvOv5093i	MicPAO3	HF969497	52	HF969747	1	A1_23	J. F. Marques
					PmAvOv5094i	MicPAO4	HF969498	90	HF969748	57	A1_60	J. F. Marques
					PmAvOv5095i	MicPAO5	HF969499	91	HF969749	58	NE9	J. F. Marques
					PmAvOv5096i	MicPAO6	HF969500	92	HF969750	59	A1_61	J. F. Marques
					PmAvOv5098i	MicPAO8	HF969501	51	HF969751	1	A1_52	J. F. Marques
					PmAvOv5099i	MicPAO9	HF969502	93	HF969752	60	A1_62	J. F. Marques
					PmAvOv50910i	MicPAO10	HF969503	21	HF969753	57	A1_5	J. F. Marques
					PmAvO50911i	MicPAO11	HF969504	51	HF969754	1	A1_52	J. F. Marques
					PmAvOv50912i	MicPAO12	HF969505	94	HF969755	1	A1_63	J. F. Marques
					PmAvOv50913i	MicPAO13			HF969756	61		J. F. Marques
					PmAvOv50914i	MicPAO14	HF96506	95	HF969757	62	A1_64	J. F. Marques
					PmAvOv50915i	MicPAO15	HF96507	52	HF969758	1	A1_23	J. F. Marques
				27. Southern Arm	PmAvS5091sp	MicPAS1	HF96508	79	HF969759	46	A1_48	J. F. Marques
					PmAvS5092sp	MicPAS2	HF96509	80	HF969760	1	A1_49	J. F. Marques
					PmAvS5093sp	MicPAS3	HF96510	52	HF969761	1	A1_23	J. F. Marques
					PmAvS5094sp	MicPAS4	HF96511	81	HF969762	47	A1_50	J. F. Marques
					PmAvS5095sp	MicPAS5	HF96512	52	HF969763	1	A1_23	J. F. Marques
					PmAvS5096sp	MicPAS6	HF96513	82	HF969764	48	NE8	J. F. Marques
					PmAvS5097sp	MicPAS7	HF96514	83	HF969765	49	M41	J. F. Marques
					PmAvS5098sp	MicPAS8	HF96515	84	HF969766	50	A1_51	J. F. Marques
					PmAvS5099sp	MicPAS9	HF96516	51	HF969767	1	A1_52	J. F. Marques
					PmAvS50910sp	MicPAS10	HF96517	85	HF969768	51	A1_53	J. F. Marques
					PmAvS50911sp	MicPAS11	HF96518	86	HF969769	1	A1_54	J. F. Marques
					PmAvS50912sp	MicPAS12	HF96519	87	HF969770	1	A1_55	J. F. Marques
					PmAvS50913sp	MicPAS13	HF96520	88	HF969771	52	A1_56	J. F. Marques
					PmAvS50914sp	MicPAS14			HF969772	53		J. F. Marques
					PmAvS50915sp	MicPAS15	HF96521	89	HF969773	54	A1_57	J. F. Marques
			28. Mira		PmM10091	MicPM1	HF96522	71	HF969774	39	A1_42	J. F. Marques
					PmM10092	MicPM2	HF96523	64	HF969775	40	A1_43	J. F. Marques
			Tagus Estuary	29. Alcochete	PmTAl5091m	MicPTA1	HF96524	44	HF969776	12	A1_15	J. F. Marques
					PmTAl5092m	MicPTA2			HF969777	13		J. F. Marques
					PmTAl5093m	MicPTA3	HF969525	45	HF969778	11	A1_16	J. F. Marques
					PmTAl5094m	MicPTA4	HF969526	46	HF969779	14	A1_17	J. F. Marques
					PmTAl5095m	MicPTA5	HF969527	47	HF969780	15	A1_18	J. F. Marques
					PmTAl5096m	MicPTA6	HF969528	48	HF969781	16	A1_19	J. F. Marques
					PmTAl5097m	MicPTA7	HF969529	49	HF969782	17	A2_4	J. F. Marques
					PmTAl5098m	MicPTA8	HF969530	50	HF969783	18	A1_20	J. F. Marques
					PmTAl5099m	MicPTA9	HF969531	43	HF969784	19	A1_21	J. F. Marques
					PmTAl50910m	MicPTA10	HF969532	42	HF969785	9	A2_3	J. F. Marques
					PmTAl50911m	MicPTA11	HF969533	51	HF969786	20	A1_22	J. F. Marques
					PmTAl50912m	MicPTA12	HF969534	52	HF969787	1	A1_23	J. F. Marques
					PmTAl50913m	MicPTA13	HF969535	52	HF969788	21	A1_24	J. F. Marques
					PmTAl50914m	MicPTA14	HF969536	47	HF969789	22	A1_25	J. F. Marques
					PmTAl50915m	MicPTA15	HF969537	52	HF969790	1	A1_23	J. F. Marques
				30. Villa Franca	PmTvfx5091m	MicPTVF1			HF969791	1		J. F. Marques
					PmTvfx5092m	MicPTVF2	HF969538	37	HF969792	1	A1_11	J. F. Marques
					PmTvfx5093m	MicPTVF3	HF969539	38	HF969793	2	NE5	J. F. Marques
					PmTvfx5094m	MicPTVF4	HF969540	39	HF969794	3	A1_12	J. F. Marques
					PmTvfx5095m	MicPTVF5	HF969541	40	HF969795	4	NE6	J. F. Marques
					PmTvfx5096m	MicPTVF6			HF969796	1		J. F. Marques
					PmTvfx5097m	MicPTVF7			HF969797	5		J. F. Marques
					PmTvfx5098m	MicPTVF8	HF969542	41	HF969798	6	A1_13	J. F. Marques
					PmTvfx50910m	MicPTVF10	HF969543	42	HF969799	7	A2_2	J. F. Marques
					PmTvfx50911m	MicPTVF11	HF969544	43	HF969800	1	A1_14	J. F. Marques
					PmTvfx50912m	MicPTVF12			HF969801	1		J. F. Marques
					PmTvfx50913m	MicPTVF13			HF969802	8		J. F. Marques
					PmTvfx50914m	MicPTVF14	HF969545	42	HF969803	9	A2_3	J. F. Marques
					PmTvfx50915m	MicPTVF15			HF969804	10		J. F. Marques
					PmTvfx50916m	MicPTVF16			HF969805	11		J. F. Marques
			Sado River	31. Carrasqueira	PmSC7091su	MicPSCsu1	HF969546	65	HF969806	36	M39	J. F. Marques
					PmSC7092su	MicPSCsu2			HF969807	37		J. F. Marques
					PmSC7091sp	MicPSCsp1	HF969547	66	HF969808	38	A1_37	J. F. Marques
					PmSC7091i	MicPSCi1	HF969548	67	HF969809	32	A1_38	J. F. Marques
					PmSC7093i	MicPSCi3	HF969549	68	HF969810	33	M40	J. F. Marques
					PmSC7096i	MicPSCi6	HF969550	63	HF969811	34	A1_39	J. F. Marques
					PmSC7097i	MicPSCi7	HF969551	69	HF969812	25	A1_40	J. F. Marques
					PmSC7099i	MicPSCi9	HF969552	70	HF969813	35	A1_41	J. F. Marques
				32. Gambia	PmSG7093sp	MicPSGsp3	HF969553	53	HF969814	23	A1_26	J. F. Marques
					PmSG7094sp	MicPSGsp4	HF969554	54	HF969815	24	A1_27	J. F. Marques
					PmSG7095sp	MicPSGsp5	HF969555	55	HF969816	25	A1_28	J. F. Marques
					PmSG7096sp	MicPSGsp6			HF969817	26		J. F. Marques
					PmSG7097sp	MicPSGsp7	HF969556	56	HF969818	27	A1_29	J. F. Marques
					PmSG708sp	MicPSGsp8	HF969557	57	HF969819	1	A1_30	J. F. Marques
					PmSG7091i	MicPSGi1	HF969558	58	HF969820	28	A1_31	J. F. Marques
					PmSG7092i	MicPSGi2	HF969559	59				J. F. Marques
					PmSG7093i	MicPSGi3	HF969560	60	HF969821	25	A1_32	J. F. Marques
					PmSG7094i	MicPSGi4	HF969561	61	HF969822	29	A1_33	J. F. Marques
					PmSG7095i	MicPSGi5	HF969562	62	HF969823	1	A1_34	J. F. Marques
					PmSG7096i	MicPSGi6	HF969563	63	HF969824	30	A1_35	J. F. Marques
					PmSG7097i	MicPSGi7	HF969564	64	HF969825	31	A1_36	J. F. Marques
					PmSG7098i	MicPSGi8	HF969565	52				J. F. Marques
			33. Guadiana River		PmG10091	MicPG1	HF969566	72	HF969826	41	A1_44	J. F. Marques
					PmG10092	MicPG2	HF969567	73	HF969827	42	NE7	J. F. Marques
					PmG10093	MicPG3	HF969568	74				J. F. Marques
					PmG10094	MicPG4	HF969569	75	HF969828	43	A1_45	J. F. Marques
					PmG10095	MicPG5	HF969570	75				J. F. Marques
					PmG10096	MicPG6	HF969571	76	HF969829	44	A1_46	J. F. Marques
					PmG10097	MicPG7	HF969572	77				J. F. Marques
					PmG10098	MicPG8	HF969573	78	HF969830	45	A1_47	J. F. Marques
P. marmoratus	France	Golfe du Lion	Salses-Leucate Lagoon	Salses	G2957	MarSa114	HF969574		HF969831			ISEM
					G2960	MarSa115	HF969575		HF969832			ISEM
				South	G2554	MarSal117	HF969576		HF969833			ISEM
					G2557	MarSal119	HF969577		HF969834			ISEM
			La Palme Lagoon		G2407	MarPal132	HF969578		HF969835			ISEM
			Thau Lagoon	ULM spot	G2904	MarTh144	HF969579		HF969836			ISEM
					G2905	MarTh145	HF969580		HF969837			ISEM
				Mèze	G2848	MarTh161	HF969581		HF969838			ISEM
					G2849	MarTh162	HF969582		HF969839			ISEM
	Italy	Adriatic	Venezia Lagoon	Venezia	G1579	MarVen174	HF969583		HF969840			ISEM
inutus	Finland	Baltic Sea	Brick Factory Bay		G3152	MinFin264			HF969841			M. Hardman
					G3157	MinFin270	HF969584		HF969842			M. Hardman
					G3159	MinFin272	HF969585		HF969843			M. Hardman
					G3160	MinFin273	HF969586		HF969844			M. Hardman
					G3161	MinFin274	HF969587		HF969845			M. Hardman
					G3162	MinBer275	HF969588		HF969846			L. Brosse

P. n	France	Golfe du Lion	Berre Lagoon		G3164	MinBer277	HF969589	HF969847	L. Brosse
					G3185	MinBer283	HF969590	HF969848	L. Brosse
					G3186	MinBer284	HF969591	HF969849	L. Brosse
					G3188	MinBer286	HF969592	HF969850	L. Brosse
	Italy	Adriatic	Venezia Lagoon	Venezia	G0563	MinVen344	HF969593	HF969851	ISEM
P. lozanoi	Belgium	North Sea	Voordelta and Ostend			Loz1	AY033049	EU737078	Stefanni & Thorley (2003); Larmuseau *et al*. (2009)
						Loz2	AY033050	EU737089	Stefanni & Thorley (2003); Larmuseau *et al*. (2009)
			Voordelta			Loz3	AY033051		Stefanni & Thorley (2003)
						Loz4	AY033052		Stefanni & Thorley (2003)
						Loz5	AY033053		Stefanni & Thorley (2003)
	France	Atlantic	Garonne Mouth	Gironde estuary		Loz6		EU737080	Larmuseau *et al*. (2009)
P. canestrini	Italy	Adriatic	Venezia Lagoon	Venezia	G0559	CanVen340	HF969594		ISEM
					G0560	CanVen341	HF969595		ISEM
					G0561	CanVen342	HF969596		ISEM
					G0562	CanVen343	HF969597		ISEM
					G1594	CanVen350	HF969598		ISEM
					G1595	CanVen351	HF969599		ISEM
					G1598	CanVen352	HF969600		ISEM
					G1599	CanVen353	HF969601	HF969852	ISEM
					G1600	CanVen354	HF969602	HF969853	ISEM
P. tortonesei	Tunisia	Mediterranean Sea	Bizerta Lagoon		G3066	Torto1	HF969603	HF969854	ISEM
					G3067	Torto2	HF969604	HF969855	ISEM
					G3068	Torto3	HF969605	HF969856	ISEM
					G3069	Torto4	HF969606	HF969857	ISEM
			Southern Tunis Lagoon		G3071	Torto5	HF969607	HF969858	ISEM
					G3072	Torto6	HF969608	HF969859	ISEM
					G3073	Torto7	HF969609	HF969860	ISEM
					G3074	Torto8	HF969610	HF969861	ISEM
	Italy	Mediterranean Sea	Sicilia	Palermo	G2372	Torto9	HF969611		ISEM
Knipowitschia panizzae	Italy	Adriatic	Venezia Lagoon	Venezia	G1544	Kpan355	HF969612		ISEM
K. caucasica						Kcau		FJ526796	Neilson & Stepien (2009)

[a]**References**

Larmuseau *et al*. (2009) Distributional and demographic consequences of Pleistocene climate fluctuations for a marine demersal fish in the north-eastern Atlantic. Journal of Biogeography, 36, 1138-1151.

Neilson & Stepien (2009) Escape from the Ponto-Caspian: evolution and biogeography of an endemic goby species flock (Benthophilinae: Gobiidae: Teleostei). Molecular Phylogenetics and Evolution, 52, 84-102.

Stefanni & Thorley (2003) Mitochondrial DNA phylogeography reveals the existence of an Evolutionarily Significant Unit of the sand goby *Pomatoschistus minutus* in the Adriatic (Eastern Mediterranean). Molecular Phylogenetics and Evolution, 28, 601-609.
