# Supplementary material for: New Light on the Evolutionary History of the Common Goby (Pomatoschistus microps) with an Emphasis on Colonization Processes in the Mediterranean Sea
Source: PLoS One. 2014 Mar 19;9(3):e91576. doi: 10.1371/journal.pone.0091576 (PMC3960122; doi:10.1371/journal.pone.0091576)
Supplement: Table S2 — Alternative topologies non-significantly different (5% confidence level) including the four lineages of Pomatoschistus microps. (PDF) [file pone.0091576.s006.pdf]

Topologies	-lnL	SE	P>0.05
1. ((Med,(Atl1,(Atl2,Out))),NEur)	1883.50257	(best)	
2. ((Med,NEur),(Atl1,(Atl2,Out)))	1883.50257	0.00000	1.000
3. (Med,((Atl1,(Atl2,Out)),NEur))	1883.50257	0.00000	0.993
4. (((Med,NEur),Atl1),(Atl2,Out))	1883.50257	0.00000	0.984
5. (((Med,NEur),(Atl2,Out)),Atl1)	1883.50257	0.00000	0.978
6. ((((Med,NEur),Atl1),Atl2),Out)	1883.50257	0.00000	0.966
7. ((((Med,NEur),Atl1),Out),Atl2)	1883.50257	0.00000	0.961
8. (((Med,(Atl1,NEur)),Atl2),Out)	1883.75170	0.24913	0.814
9. (((Med,(Atl1,NEur)),Out),Atl2)	1883.75170	0.24913	0.814
10. ((Med,(Atl1,NEur)),(Atl2,Out))	1883.75170	0.24913	0.814
11. (((Med,(Atl2,Out)),Atl1),NEur)	1883.75170	0.24913	0.814
12. (((Med,(Atl2,Out)),NEur),Atl1)	1883.75170	0.24913	0.814
13. ((Med,(Atl2,Out)),(Atl1,NEur))	1883.75170	0.24913	0.814
14. (Med,((Atl1,NEur),(Atl2,Out)))	1883.75170	0.24913	0.814
15. (((Med,Atl1),(Atl2,Out)),NEur)	1886.42489	2.92231	0.623
16. ((((Med,Atl1),NEur),Atl2),Out)	1886.42489	2.92231	0.623
17. ((((Med,Atl1),NEur),Out),Atl2)	1886.42489	2.92231	0.623
18. (((Med,Atl1),NEur),(Atl2,Out))	1886.42489	2.92231	0.623
19. ((Med,Atl1),((Atl2,Out),NEur))	1886.42489	2.92231	0.623
20. ((Med,((Atl2,Out),NEur)),Atl1)	1886.42489	2.92231	0.623
21. (Med,(Atl1,((Atl2,Out),NEur)))	1886.42489	2.92231	0.623
22. ((((Med,NEur),Out),Atl1),Atl2)	1887.26021	3.75764	0.572
23. ((((Med,NEur),Out),Atl2),Atl1)	1887.26021	3.75764	0.572
24. ((Med,((Atl1,Atl2),Out)),NEur)	1887.26021	3.75764	0.572
25. (((Med,NEur),(Atl1,Atl2)),Out)	1887.26021	3.75764	0.572
26. (((Med,NEur),Out),(Atl1,Atl2))	1887.26021	3.75764	0.572
27. ((Med,NEur),((Atl1,Atl2),Out))	1887.26021	3.75764	0.572
28. (Med,(((Atl1,Atl2),Out),NEur))	1887.26021	3.75764	0.572
29. (((Med,(NEur,Out)),Atl1),Atl2)	1887.42792	3.92535	0.553
30. (((Med,(NEur,Out)),Atl2),Atl1)	1887.42792	3.92535	0.553
31. (((Med,(Atl1,Atl2)),NEur),Out)	1887.42792	3.92535	0.553
32. (((Med,(Atl1,Atl2)),Out),NEur)	1887.42792	3.92535	0.553
33. ((Med,(Atl1,Atl2)),(NEur,Out))	1887.42792	3.92535	0.553
34. ((Med,(NEur,Out)),(Atl1,Atl2))	1887.42792	3.92535	0.553
35. (Med,((Atl1,Atl2),(NEur,Out)))	1887.42792	3.92535	0.553
36. (((Med,NEur),(Atl1,Out)),Atl2)	1888.65551	5.15294	0.486

Topologies	-lnL	SE	P>0.05
37. ((((Med,NEur),Atl2),Atl1),Out)	1888.65718	5.15460	0.486
38. ((((Med,NEur),Atl2),Out),Atl1)	1888.65023	5.14766	0.486
39. (((Med,NEur),Atl2),(Atl1,Out))	1888.65586	5.15329	0.486
40. ((Med,((Atl1,Out),Atl2)),NEur)	1888.65553	5.15295	0.486
41. ((Med,NEur),((Atl1,Out),Atl2))	1888.65552	5.15295	0.486
42. (Med,(((Atl1,Out),Atl2),NEur))	1888.65555	5.15298	0.486
43. ((Med,(Atl1,(NEur,Out))),Atl2)	1888.24505	4.74248	0.462
44. ((((Med,Atl2),Atl1),NEur),Out)	1888.24505	4.74248	0.462
45. ((((Med,Atl2),Atl1),Out),NEur)	1888.24505	4.74248	0.462
46. (((Med,Atl2),Atl1),(NEur,Out))	1888.24505	4.74248	0.462
47. (((Med,Atl2),(NEur,Out)),Atl1)	1888.24505	4.74248	0.462
48. ((Med,Atl2),(Atl1,(NEur,Out)))	1888.24505	4.74248	0.462
49. (Med,((Atl1,(NEur,Out)),Atl2))	1888.24505	4.74248	0.462
50. ((((Med,Out),NEur),Atl1),Atl2)	1888.81803	5.31545	0.441
51. ((((Med,Out),NEur),Atl2),Atl1)	1888.81803	5.31545	0.441
52. (((Med,Out),(Atl1,Atl2)),NEur)	1888.81803	5.31545	0.441
53. (((Med,Out),NEur),(Atl1,Atl2))	1888.81803	5.31545	0.441
54. ((Med,((Atl1,Atl2),NEur)),Out)	1888.81803	5.31545	0.441
55. ((Med,Out),((Atl1,Atl2),NEur))	1888.81803	5.31545	0.441
56. (Med,(((Atl1,Atl2),NEur),Out))	1888.81803	5.31545	0.441
57. (((Med,Out),(Atl1,NEur)),Atl2)	1890.06439	6.56182	0.346
58. ((((Med,Out),Atl2),Atl1),NEur)	1890.06439	6.56182	0.346
59. ((((Med,Out),Atl2),NEur),Atl1)	1890.06439	6.56182	0.346
60. (((Med,Out),Atl2),(Atl1,NEur))	1890.06439	6.56182	0.346
61. ((Med,((Atl1,NEur),Atl2)),Out)	1890.06439	6.56182	0.346
62. ((Med,Out),((Atl1,NEur),Atl2))	1890.06439	6.56182	0.346
63. (Med,(((Atl1,NEur),Atl2),Out))	1890.06439	6.56182	0.346
64. ((Med,((Atl1,Out),NEur)),Atl2)	1890.09355	6.59097	0.338
65. ((((Med,Atl2),Out),Atl1),NEur)	1890.09207	6.58949	0.338
66. (((Med,Atl2),(Atl1,Out)),NEur)	1890.09355	6.59097	0.338
67. ((((Med,Atl2),NEur),Atl1),Out)	1890.09355	6.59097	0.338
68. ((((Med,Atl2),NEur),Out),Atl1)	1890.09355	6.59097	0.338
69. ((((Med,Atl2),Out),NEur),Atl1)	1890.09207	6.58949	0.338
70. (((Med,Atl2),NEur),(Atl1,Out))	1890.09355	6.59097	0.338
71. ((Med,Atl2),((Atl1,Out),NEur))	1890.09355	6.59097	0.338
72. (Med,(((Atl1,Out),NEur),Atl2))	1890.09355	6.59097	0.338
73. ((Med,((Atl1,NEur),Out)),Atl2)	1890.08519	6.58261	0.337
74. (((Med,Atl2),(Atl1,NEur)),Out)	1890.08519	6.58261	0.337

Topologies	-lnL	SE	P>0.05
75. (((Med,Atl2),Out),(Atl1,NEur))	1890.08519	6.58261	0.337
76. ((Med,Atl2),((Atl1,NEur),Out))	1890.08519	6.58261	0.337
77. (Med,(((Atl1,NEur),Out),Atl2))	1890.08519	6.58261	0.337
78. ((((Med,Atl1),Atl2),NEur),Out)	1890.92309	7.42052	0.281
79. ((((Med,Atl1),Atl2),Out),NEur)	1890.92309	7.42052	0.281
80. (((Med,Atl1),Atl2),(NEur,Out))	1890.92309	7.42052	0.281
81. (((Med,Atl1),(NEur,Out)),Atl2)	1890.92309	7.42052	0.281
82. ((Med,Atl1),(Atl2,(NEur,Out)))	1890.92309	7.42052	0.281
83. ((Med,(Atl2,(NEur,Out))),Atl1)	1890.92309	7.42052	0.281
84. (Med,(Atl1,(Atl2,(NEur,Out))))	1890.92309	7.42052	0.281
85. ((((Med,Atl1),Out),Atl2),NEur)	1892.76540	9.26283	0.192
86. (((Med,(Atl1,Out)),Atl2),NEur)	1892.76540	9.26283	0.192
87. ((((Med,Atl1),Out),NEur),Atl2)	1892.76540	9.26283	0.192
88. (((Med,(Atl1,Out)),NEur),Atl2)	1892.76540	9.26283	0.192
89. (((Med,Atl1),(Atl2,NEur)),Out)	1892.76540	9.26283	0.192
90. (((Med,Atl1),Out),(Atl2,NEur))	1892.76540	9.26283	0.192
91. ((Med,(Atl1,Out)),(Atl2,NEur))	1892.76540	9.26283	0.192
92. ((Med,Atl1),((Atl2,NEur),Out))	1892.76540	9.26283	0.192
93. (((Med,(Atl2,NEur)),Atl1),Out)	1892.76540	9.26283	0.192
94. (((Med,(Atl2,NEur)),Out),Atl1)	1892.76540	9.26283	0.192
95. ((Med,((Atl2,NEur),Out)),Atl1)	1892.76540	9.26283	0.192
96. ((Med,(Atl2,NEur)),(Atl1,Out))	1892.76540	9.26283	0.192
97. (Med,((Atl1,Out),(Atl2,NEur)))	1892.76540	9.26283	0.192
98. (Med,(Atl1,((Atl2,NEur),Out)))	1892.76540	9.26283	0.192
99. ((((Med,Out),Atl1),Atl2),NEur)	1892.74955	9.24698	0.189
100. ((((Med,Out),Atl1),NEur),Atl2)	1892.74955	9.24698	0.189
101. (((Med,Out),Atl1),(Atl2,NEur))	1892.74955	9.24698	0.189
102. (((Med,Out),(Atl2,NEur)),Atl1)	1892.74955	9.24698	0.189
103. ((Med,(Atl1,(Atl2,NEur))),Out)	1892.74955	9.24698	0.189
104. ((Med,Out),(Atl1,(Atl2,NEur)))	1892.74955	9.24698	0.189
105. (Med,((Atl1,(Atl2,NEur)),Out))	1892.74955	9.24698	0.189
