# Supplementary material for: New Light on the Evolutionary History of the Common Goby (Pomatoschistus microps) with an Emphasis on Colonization Processes in the Mediterranean Sea
Source: PLoS One. 2014 Mar 19;9(3):e91576. doi: 10.1371/journal.pone.0091576 (PMC3960122; doi:10.1371/journal.pone.0091576)
Supplement: Table S3 — Distribution and number of concatenated control region and cytochrome b gene haplotypes per locality (Sc, Scotland; Ire, Ireland). The haplotype number is in the first column, while the locality number (Fig. 1 and Table S1) is in the first row. (PDF) [file pone.0091576.s007.pdf]

	Sc	Ire	France (Atlantic)					France (Golfe du Lion)									Spain (Ebro)			Portugal				
	1	2	3	4	5	6	7	8	9	10	11	12	13	14 17	18	19	20 21	22 23	24 25	26 27	28	29 30	31 32	33
A1_1							1					1												
A1_2												1												
M1														4	1									
M2														3	1									
M3														1										
M4														1										
M5														2										
M6											1			2										
M7														1										
M8											1			1										
M9														1										
M10														1										
M11														1			7	3	3					
M12										1	1				1									
M13										1														
M14										1														
M15										1														
M16								1		1			2											
M17																			1					
M18																		2	1					
M19																			1					
M20																			1					
M21																	2	3	1					
M22																	1							
M23																		1						
M24																		1						
M25											1													
M26											1													
M27								1					1											
M28													2											
M29								1																
M30															1									
M31															1									
M32															1									
M33															1									
M34															1									
M35																1								
M36																1								
M37																1								
A1_3					1																			
M38					1																			
A2_1					1																			
A1_4					1																			
A1_5					1															1				
A1_6					1																			
A1_7					1																			
A1_8					1																			

	Sc	Ire	France (Atlantic)					France (Golfe du Lion)									Spain (Ebro)			Portugal				
	1	2	3	4	5	6	7	8	9	10	11	12	13	14<br>17	18	19	20<br>21	22<br>23	24<br>25	26<br>27	28	29<br>30	31<br>32	33
A1_9					1																			
NE1					1																			
A1_10					1																			
NE2	2																							
NE3	1																							
NE4	1																							
A1_11																						1		
NE5																						1		
A1_12																						1		
NE6																						1		
A1_13																						1		
A2_2																						1		
A1_14																						1		
A2_3																						2		
A1_15																						1		
A1_16																						1		
A1_17																						1		
A1_18																						1		
A1_19																						1		
A2_4																						1		
A1_20																						1		
A1_21																						1		
A1_22																						1		
A1_23																				4		2		
A1_24																						1		
A1_25																						1		
A1_26																							1	
A1_27																							1	
A1_28																							1	
A1_29																							1	
A1_30																							1	
A1_31																							1	
A1_32																							1	
A1_33																							1	
A1_34																							1	
A1_35																							1	
A1_36																							1	
M39																							1	
A1_37																							1	
A1_38																							1	
M40																							1	
A1_39																							1	
A1_40																							1	
A1_41																							1	
A1_42																					1			
A1_43																					1			
A1_44																								1
NE7																								1
A1_45																								1

	Sc	Ire	France (Atlantic)					France (Golfe du Lion)									Spain (Ebro)			Portugal				
	1	2	3	4	5	6	7	8	9	10	11	12	13	14 17	18	19	20 21	22 23	24 25	26 27	28	29 30	31 32	33
A1_46																								1
A1_47																								1
A1_48																				1				
A1_49																				1				
A1_50																				1				
NE8																				1				
M41																				1				
A1_51																				1				
A1_52																				3				
A1_53																				1				
A1_54																				1				
A1_55																				1				
A1_56																				1				
A1_57																				1				
A1_58																				1				
A1_59																				1				
A1_60																				1				
NE9																				1				
A1_61																				1				
A1_62																				1				
A1_63																				1				
A1_64																				1				
A1_65				1																				
A1_66				1																				
NE10		2																						
A1_67		1																						
NE11		1																						
NE12		1																						
NE13		1																						
NE14		1																						
NE15		1																						
NE16		1																						
NE17		1																						
A1_68						1																		
A1_69						1																		
A1_70						1																		
M42						1																		
NE18						1																		
A1_71						1																		
NE19						1																		
A1_72						1																		
A1_73						1																		
A1_74						1																		
A1_75						1																		
NE20			1																					
NE21			1																					
A1_76			1																					
A1_77			1																					
